# Emerging zero-dimensional to four-dimensional biomaterials for bone regeneration

**DOI:** 10.1186/s12951-021-01228-1

**Published:** 2022-01-06

**Authors:** Haoyu Fang, Daoyu Zhu, Qianhao Yang, Yixuan Chen, Changqing Zhang, Junjie Gao, Youshui Gao

**Affiliations:** 1 Department of Orthopedic Surgery, Shanghai Jiao Tong University Affiliated Sixth People’s Hospital, Shanghai, China; 2 Ningbo Institute of Life and Health Industry, University of Chinese Academy of Science, Ningbo, Zhejiang China

**Keywords:** Zero/one/two/three/four-dimensional biomaterial, Tissue engineering, Bone regeneration

## Abstract

Bone is one of the most sophisticated and dynamic tissues in the human body, and is characterized by its remarkable potential for regeneration. In most cases, bone has the capacity to be restored to its original form with homeostatic functionality after injury without any remaining scarring. Throughout the fascinating processes of bone regeneration, a plethora of cell lineages and signaling molecules, together with the extracellular matrix, are precisely regulated at multiple length and time scales. However, conditions, such as delayed unions (or nonunion) and critical-sized bone defects, represent thorny challenges for orthopedic surgeons. During recent decades, a variety of novel biomaterials have been designed to mimic the organic and inorganic structure of the bone microenvironment, which have tremendously promoted and accelerated bone healing throughout different stages of bone regeneration. Advances in tissue engineering endowed bone scaffolds with phenomenal osteoconductivity, osteoinductivity, vascularization and neurotization effects as well as alluring properties, such as antibacterial effects. According to the dimensional structure and functional mechanism, these biomaterials are categorized as zero-dimensional, one-dimensional, two-dimensional, three-dimensional, and four-dimensional biomaterials. In this review, we comprehensively summarized the astounding advances in emerging biomaterials for bone regeneration by categorizing them as zero-dimensional to four-dimensional biomaterials, which were further elucidated by typical examples. Hopefully, this review will provide some inspiration for the future design of biomaterials for bone tissue engineering.

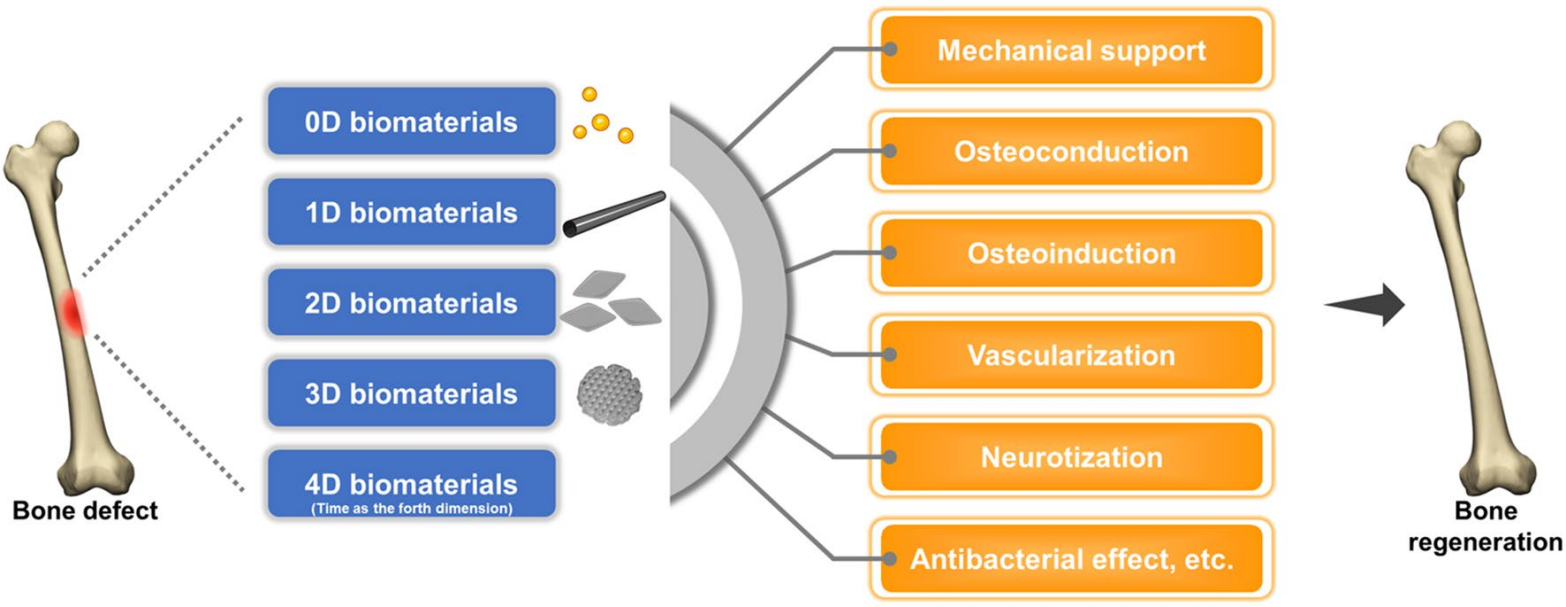

## Introduction

Critical-sized bone defects resulting from trauma and other diseases remain a major challenge for orthopedic surgeons and appeal to the development of suitable bone grafts. Autologous bone grafts, which are generally regarded as the “gold standard” treatment for critical-sized bone defects, suffer from several disadvantages, such as quite limited source, prolonged operative time, and harvest site morbidity [1]. Therefore, artificial bone grafts emerge as alternative choices for bone tissue regeneration. A variety of biomaterials (e.g., polymer scaffolds, bioactive glasses, and hydrogels) have been intensively investigated as promising candidates for the effective reconstruction of bone defects.

The natural process of bone repair comprises a series of precisely-controlled synchronous and sequential events, which involve multiple cell populations, various biomolecules, extracellular matrices, and active interactions between all the components within the bone microenvironment [2]. To coordinate with the sophisticated process of bone regeneration, several characteristics and key parameters must be taken into account when designing biomaterials for bone tissue regeneration: (1) great biocompatibility and biodegradability to avoid potential inflammatory responses and rejection reactions; (2) proper surface properties to facilitate cell attachment and ingrowth, namely osteoconduction [3]; (3) a hierarchical interconnected porous structure to enable the ingrowth of vessels and neurons, and to permit the transport of nutrients and metabolites; (4) the capability of inducing pluripotent cells into osteogenic cell lineages via a process referred to as osteoinduction [3]; and (5) sufficient mechanical strength and structural integrity to sustain mechanical stress during bone remodeling.

In hopes of elucidating the correlation between material geometry and cell fate, we classified the biomaterials within the field of bone tissue regeneration according to the dimensional geometry and size in each dimension [i.e., number of dimensions that are confined to the nanoscale (< 100 nm)]. Specifically, nanoparticles with all three dimensions strictly confined to the nanoscale are defined as zero-dimensional biomaterials; one-dimensional biomaterials refer to nanomaterials with only two dimensions confined to the nanoscale; two-dimensional biomaterials have only one dimension within the nanoscale range; and all dimensions of three-dimensional biomaterials are greater than the nanoscale [4]. Four-dimensional biomaterials, which have emerged in recent years, integrate the concept of time as the fourth dimension [5].

## Zero-dimensional biomaterials

As mentioned above, the classification system elucidated in this article is generally based on the dimensional geometry and size in each dimension [i.e., number of dimensions that are strictly confined to the nanoscale (< 100 nm)].

Nanoparticles with all three dimensions strictly confined to the nanoscale are defined as zero-dimensional biomaterials [4]. Due to the high surface-to-volume ratio, zero-dimensional biomaterials exhibit several distinct physicochemical properties.

### Carbon-based zero-dimensional biomaterials

During the past few decades, a variety of carbon-based zero-dimensional biomaterials have been fabricated, among which fullerene, nanodiamonds, and carbon dots are the most widely discussed.

#### Fullerene

First discovered in 1985, fullerene (C_60_) has attracted considerable attention given its fascinating crystal structure, electronic properties, and physicochemical characteristics [6–8]. Geim [9] described fullerene as a wrapped form of a two-dimensional graphene sheet. Unlike graphene, however, the 60 carbon atoms contained in fullerene are arranged in a closed spherical form, which explains its significant diversity during aggregation [10, 11]. In other words, this unique morphological structure enables fullerene to assemble into one-, two-, and three-dimensional structures depending on the type of solvent mixture [12–14]. In 2015, Krishnan et al. [15] fabricated aligned fullerene nanowhiskers (C_60_NWs) via the vortex motion method, which were subsequently transferred onto glass substrates as a scaffold for cell culture. It turned out that MG-63 cells were highly oriented in accordance with the axis of the aligned C_60_NWs. In addition, it has been reported that polyhydroxylated fullerene not only supported the proliferation of human adipose-derived stem cells (hADSCs), but also facilitated the process of osteogenic differentiation and biomineralization, of which the mechanism has not been fully explored yet [16]. The production of cytotoxic reactive oxygen species (ROS) by photoexcited fullerene, however, may hinder the further application of fullerene as a friendly biomaterial for bone regeneration [17].

#### Nanodiamonds

Another carbon-based nanomaterial that deserves attention is nanodiamonds (NDs), which are defined as nanocrystalline diamonds with a diameter of 5–8 nm. Due to the high surface-to-volume ratio, NDs, which are chemically inert, could be easily surface-functionalized with a variety of chemically reactive moieties such as -COOH. Many efforts have been made to apply NDs to biomaterial scaffolds in the last two decades, given the unique and superior properties of NDs, such as extreme hardness, superior mechanical strength, high thermal conductivity, great chemical stability and surface reactivity, and excellent biocompatibility [18].

A study showed that nanostructured nanocrystalline diamond (NCD) films provide better support for the adhesion, metabolic activity and osteogenic differentiation of MG-63 cells compared to the control polystyrene culture dish [19]. Astonishingly, even a small quantity (e.g., 10% wt) of octadecylamine-functionalized nanodiamond (ND-ODA) incorporated in poly(l-lactic acid) (PLLA) could result in remarkable potentiation in the mechanical performance of the composite scaffold (e.g., Young’s modulus, strain at failure, tensile strength, and hardness) [20, 21]. Furthermore, due to the high surface reactivity of NDs, ND-ODA/PLLA scaffolds interact with a variety of moieties and ions in the simulated body fluid and facilitate the deposition of bone-like apatite, which showed excellent mineralization capability and could be of great benefit for bone regrowth [21]. The addition of NDs into poly(lactic-co-glycolic acid) (PLGA) nanofibrous scaffolds remarkably promoted its mechanical performance in rupture tests, and the composite scaffold also exhibited great biocompatibility to enable the adhesion, spreading, and proliferation of MG-63 cells without evoking considerable inflammatory reactions of RAW 264.7 macrophages and MG-63 cells [22]. In addition, intrinsic fluorescence emitted by NDs under certain wavelengths of light could be of great value for in vivo monitoring of ND-ODA/PLLA-based internal fixation devices during surgery and during the process of bone healing [20]. In summary, NDs exhibit a great diversity of fascinating properties (i.e., superior mechanical strength, great chemical stability and surface reactivity, excellent biocompatibility, favorable effects on cell proliferation and differentiation, remarkable mineralization capability, and strong intrinsic fluorescence), which makes ND-based scaffolds promising biomaterials for bone regenerative engineering.

#### Carbon dots

As emerging carbon-based zero-dimensional nanomaterials, Carbon dots (C-dots) have drawn widespread attention since their emergence [23, 24]. The most distinguishing feature of C-dots is their excitation wavelength dependent photoluminescence spectra, with emissions ranging from the visible wavelength to the near-infrared wavelength [24]. Interestingly, the photoluminescence of C-dots exhibits strong resistance to photobleaching, which makes C-dots an ideal tracer for monitoring the progress of scaffold biodegradation. In addition, several in vitro and in vivo studies have elucidated that C-dots have great biocompatibility with minimal cytotoxicity when applied at appropriate concentrations (e.g., 10 μg/ml) [25–31], whereas high concentrations of C-dots (i.e., higher than 50 μg/ml) could exert an inhibitory effect on cell proliferation [25, 27].

Numerous studies have demonstrated that C-dots alone could substantially facilitate the osteogenic differentiation of mesenchymal stem cells (MSCs), which was corroborated by enhanced ALP activity and up-regulation of osteogenic gene markers [e.g., runt-related transcription factor 2 (Runx2), osteopontin (OPN), bone sialoprotein (BSP), and osteocalcin (OCN)] [25–28]. On the other hand, the addition of C-dots to hydroxyapatite (HA) and polymers has been reported to improve the mechanical performance, osteoconductivity, and osteoinductivity of composite biomaterials [32–36]. For example, Khajuria et al. [33] found that HA nanoparticles conjugated with nitrogen-doped C-dots exhibited a more favorable influence on the proliferation, osteogenic differentiation, and calcium mineralization of MC3T3-E1 cells compared with HA nanoparticles alone, presumably via the internalization of the conjugates into osteoblasts and thus activating the bone morphogenetic protein (BMP) signaling pathway. Furthermore, this conjugate significantly accelerated bone metabolism and mineralization in the zebrafish jawbone regeneration model. However, another study discovered that doping of C-dots in calcium phosphate nanorods induced ectopic chondrogenesis rather than osteogenesis in a rat subcutaneous model, presumably by activating the HIF-α/SOX-9 signaling pathway [37].

It is also worth noting that, aside from the unique photoluminescence and osteoinductive capability, C-dots exhibit several other intriguing characteristics as biomaterials for bone regeneration. It has been reported that the superior photothermal effect made C-dots a promising candidate for osteosarcoma ablation, suppressing tumor growth or even eradicating the tumor. On the other hand, the excellent osteoinductive capability of C-dots could expedite the process of subsequent bone tissue regeneration after photothermal tumor ablation is accomplished. Moreover, chitosan/nanohydroxyapatite scaffolds possessed better antibacterial properties when doped with C-dots and irradiated under near-infrared (NIR) irradiation due to the photothermal effect of C-dots [34]. Regarding primary and metastatic tumors in the bone tissue, biomaterials with superior photothermal effect and excellent osteoinductivity would have incomparable advantages in eradicating tumors and facilitating the repairing process of critical-sized bone defects arising from tumor ablation. As for the treatment of infective bone defects (e.g., infected nonunion), biomaterials with potent antibacterial property and excellent osteoinductive capability could effectively clear the persistent bacterial infection and facilitate the reconstruction of bone defects.

### Other zero-dimensional biomaterials

Aside from carbon-based zero-dimensional nanoparticles, a variety of inorganic nanoparticles have shown incredible potential for promoting MSC proliferation as well as facilitating the process of osteogenic differentiating and biomineralization.

Metallic nanoparticles (e.g., gold nanoparticles and silver nanoparticles) and metallic oxide nanoparticles (e.g., iron oxide nanoparticles) have been reported to influence MSC fate and direct MSC differentiation toward the osteogenic lineage, mainly by causing intracellular mechanical stress. For example, Yi et al. [38] discovered that gold nanoparticles (AuNPs) co-cultured with MSCs could exert mechanical stress on stem cells to activate p38 MAPK signaling pathway and subsequently up-regulate the expression of osteogenesis-related genes, which resulted in elevated ALP activity and promoted mineralization rates. In addition, silver nanoparticles (AgNPs) encapsulated in collagen favored MSC proliferation, osteogenic differentiation, and calcium mineralization, which was corroborated in a mouse femoral fracture model [39]. As for metallic oxide nanoparticles, it has been reported that iron oxide nanoparticles (IONPs) exhibited excellent biocompatibility as well as promotive effects on MSC osteogenic differentiation and biomineralization, resulting from the activation of MAPK pathway and the subsequent up-regulation of downstream genes that are related to osteogenesis (e.g., Runx2 and BMP-2) [40]. Of note, not only can AuNPs interact with MSCs and facilitate osteogenesis, but they can also serve as cellular probes for MSC tracking in vivo. In 2016, Wan et al. [41] fabricated AuNPs@SiO_2_-TS by modifying silica-coated AuNPs with DNA Transfectin 3000 (TS), in which silica was incorporated to promote biocompatibility and TS was incorporated to enhance the cellular uptake of the composite nanoparticles. While exhibiting no adverse effect on cell viability and multi-directional differentiation capability of MSCs with concentrations lower than 100 μg/ml in vitro, AuNPs@SiO_2_-TS could be uptaken by MSCs effectively and retained in MSCs for more than 14 days. Using dual-energy computer tomography (DECT) and proper decomposition methods, MSCs labeled by AuNPs@SiO_2_-TS could be clearly visualized and distinguished from the surrounding bone tissue in a rabbit femoral bone defect model, indicating that AuNPs@SiO_2_-TS could serve as a noninvasive probe for the real-time tracking of MSCs in vivo.

In recent years, magnetic nanoparticles have also emerged as promising zero-dimensional biomaterials for bone tissue engineering. For example, Liu et al. [42] incorporated magnetic SrFe_12_O_19_ nanoparticles into bioglass/chitosan scaffolds to fabricate multi-functional hybrid scaffolds for photothermal tumor therapy and subsequent bone regeneration. The excellent photothermal conversion property of SrFe_12_O_19_ endowed the hybrid scaffolds with great efficiency in killing osteosarcoma cells, which was corroborated in a nude rat subcutaneous MNNG tumor model. On the other hand, thanks to the superior saturation magnetization and coercivity of SrFe_12_O_19_, the magnetic field produced by the hybrid scaffolds could significantly promote MSC proliferation and up-regulate the expression of osteogenesis-related genes, presumably by activating BMP-2/Smad/Runx2 signaling pathway. The exceptional bone regeneration capability of the hybrid scaffolds was also confirmed in a rat critical-sized calvarial defect model.

In summary, the high surface-to-volume ratio of zero-dimensional biomaterials endows them with exceptional properties, such as great mechanical strength, thermal conductivity, electric properties, and surface reactivity (modulating cell fate and facilitating bone mineralization). Incorporation of zero-dimensional biomaterials with other substrates could provide multifunctional platforms for the treatment of bone diseases that could cause bone defects (e.g., osteosarcoma) to serve as stepwise countermeasures to treat the aforementioned bone diseases and facilitate subsequent bone regeneration. Zero-dimensional nanoparticles are generally incorporated with other biomaterials to cater to the needs for bone defect repair, given that zero-dimensional nanoparticles alone lack the prerequisite structural integrity and stability for bone regeneration. However, the interaction mechanism between zero-dimensional biomaterials and MSCs, as well as the potential cytotoxicity of these nanoparticles, have yet to be further elucidated. Selective examples of zero-dimensional biomaterials for bone tissue engineering are briefly provided in Table 1.

**Table 1:** Selective examples of zero-dimensional biomaterials for bone tissue engineering

Biomaterial	Composite biomaterial	Modification	Fabricated into 3D scaffold?	In vitro model	In vivo model	Effect(s)	Ref.
Fullerene	–	–	–	MG-63	–	To orient cell growth	[15]
Fullerene	–	Polyhydroxylated	–	hADSCs	–	To support cell proliferation; to promote osteogenic differentiation and calcium mineralization	[16]
Nanodiamond	–	–	–	MG-63	–	To support cell adhesion, metabolic activity and osteogenic differentiation	[19]
Nanodiamond	PLLA	Octadecylamine-functionalized	Yes	7F2	–	To improve mechanical properties; to support cell proliferation and osteogenic differentiation; to facilitate calcium mineralization	[20, 21]
Nanodiamond	PLGA	–	–	MG-63	–	To improve mechanical properties; to support cell adhesion and proliferation	[22]
Carbon dots	–	–	–	rMSCs; hMSCs	–	To support cell adhesion and proliferation; to promote osteogenic differentiation and calcium mineralization	[25, 26, 28]
Carbon dots	–	Nitrogen-doped	–	rMSCs	–	To support cell adhesion and proliferation; to promote osteogenic differentiation and calcium mineralization	[27]
Carbon dots	HA + PU	–	–	MG-63	–	To improve mechanical properties; to support cell adhesion and proliferation; to promote osteogenic differentiation	[32]
Carbon dots	HA	Nitrogen-doped	–	MC3T3-E1	Zebrafish jawbone repair model	To promote cell proliferation, osteogenic differentiation, and calcium mineralization; to accelerate bone regeneration in vivo	[33]
Carbon dots	HA + Chitosan	–	Yes	rMSCs; UMR-106; *S. aureus*; *E. coli*	Rat intramuscular pouch model; Mouse subcutaneous tumor-bearing model	To promote cell adhesion and osteogenic differentiation; to accelerate bone regeneration in vivo; photothermal effect; antibacterial effect	[34]
Carbon dots	HA + CMC	–	–	MG-63	–	To support cell adhesion and proliferation; to promote osteogenic differentiation and calcium mineralization	[35]
Carbon dots	[PCL/PVA] + TCP	–	Yes	hBFPSCs	–	To support cell adhesion; to promote cell proliferation and osteogenic differentiation	[36]
AuNPs	–	–	–	mMSCs	–	To promote cell proliferation; to promote osteogenic differentiation and calcium mineralization; to inhibit adipocytic differentiation	[38]
AgNPs	Collagen	–	Yes	mMSCs	Mouse femoral fracture model	To support cell proliferation; to promote osteogenic differentiation and calcium mineralization; to accelerate bone regeneration in vivo	[39]
IONPs	–	PSC-coated	–	hMSCs	–	To support cell proliferation; to promote osteogenic differentiation and calcium mineralization	[40]
SrFe_12_O_19_ nanoparticles	Chitosan + Bioglass	–	Yes	hMSCs	Rat critical-sized calvarial defect model; rat subcutaneous tumor-bearing model	To promote cell proliferation; to promote osteogenic differentiation and calcium mineralization; to accelerate bone regeneration in vivo; photothermal effect	[42]

*hADSCs* human adipose-derived stem cells; *PLLA* poly(L-lactic acid); *PLGA* poly(lactide-co-glycolide); *HA* hydroxyapatite; *PU* polyurethane; *CMC* carboxymethyl cellulose; *PCL* poly(ε-caprolactone); *PVA* polyvinyl alcohol; *TCP* tricalcium phosphate; *MG-63* human osteosarcoma cells; *7F2* mouse bone marrow derived osteoblastic cells; *rMSCs* rat mesenchymal stem cells; *hMSCs* human mesenchymal stem cells; *MC3T3-E1* mouse preosteoblastic cells; *UMR-106* rat osteosarcoma cells; *S. aureus Staphylococcus aureus*; *E. coli Escherichia coli*; *hBFPSCs* human buccal fat pad-derived stem cells; *AuNPs* gold nanoparticles; *mMSCs* mouse mesenchymal stem cells; *AgNPs* silver nanoparticles; *IONPs* iron oxide nanoparticles; *PSC* polyglucose-sorbitol-carboxymethyether; *Ref.* references

## One-dimensional biomaterials

One-dimensional biomaterials refer to nanomaterials with only two dimensions confined to the nanoscale (< 100 nm), which can be further subdivided into nanowires, nanotubes, etc. [4]. Due to the unique morphology (e.g., high length-to-diameter ratio) and nanotopography, one-dimensional biomaterials have an extremely high degree of anisotropy, which results in various distinct properties. Moreover, numerous one-dimensional biomaterials have served as the basic building block for fabricating higher-dimensional biomaterials.

### Nanowires

Nanowires are defined as solid nanomaterials with lengths longer than 100 nm and diameters confined to the nanoscale. The length-to-diameter ratio is the key characteristic of nanowires, and this feature plays an essential role in influencing cell fates [43]. Early in 2007, the biocompatibility of the vertically aligned silicon nanowire (SiNW) array on a Si wafer was corroborated in several mammalian cell lines [44]. In 2013, it was reported that the interaction between MSCs and vertically aligned SiNW arrays on silicon substrates preferentially resulted in osteogenic and chondrogenic differentiation of MSCs, apart from the significant improvement in cell adhesion and proliferation [45]. Mechanical stimulation during MSCs-SiNW interaction triggers cytoskeletal reorganization and transiently activates Ca^2+^ channels, which subsequently initiate the Ras/Raf/MEK/ERK signaling pathway to modulate cell adhesion, proliferation, and differentiation. Furthermore, various mechanosensitive pathways (e.g., Akt, insulin, TGF-β/BMP, MAPK/ERK, integrin, and Wnt signaling pathways) also play essential roles in activating Ras/Raf/MEK/ERK cascades and initiating the process of osteogenesis and chondrogenesis when MSCs were co-cultured with the vertically aligned SiNW array [46]. In addition to SiNW, bioactive glass nanofibers (BG-NFs) [47] and nanofibers composed of poly(lactide‐co‐glycolide) and nanohydroxyapatite (PLGA/nHA-NFs) [48] have also shown great potential for the osteoinduction of MSCs.

### Nanotubes

Nanotubes are hollow cylindrical nanostructures that have drawn considerable attention due to their extraordinary mechanical, chemical, and electrical properties. The nanotube biomaterials currently used for bone regeneration mainly include titanium dioxide nanotubes (TiO_2_-NTs) and carbon nanotubes (CNTs).

#### Titanium dioxide nanotubes (TiO_2_-NTs)

The length and diameter of TiO_2_-NTs could be precisely controlled over a wide range via electrochemical anodization, which enables researchers to explore the relationship between the morphology/nanotopography of TiO_2_-NTs and stem cell fate [49]. Numerous studies have demonstrated that the diameter of vertically oriented TiO_2_-NTs on Ti substrates determines the intrinsic properties of the biomaterial (e.g., hydrophilia), thus influencing cell adhesion, spreading, proliferation, and differentiation [50–54]. Specifically, small TiO_2_-NTs (diameter ranging from 15 to 30 nm) augmented MSC adhesion and proliferation, whereas such cell activities were severely impaired on TiO_2_-NTs with a diameter greater than 50 nm [50, 52, 53]. In addition, larger TiO_2_-NTs (diameter ranging from 70 to 100 nm) could trigger MSC elongation and osteogenic differentiation, presumably by inducing cytoskeletal stress [52, 54]. Thanks to such unique diameter-dependent MSC behavior, osteogenic differentiation of MSCs could be precisely modulated by altering the diameter of vertically oriented TiO_2_-NTs on Ti substrates, offering a promising route for bone regeneration.

#### Carbon nanotubes (CNTs)

As indicated by Geim [9], carbon nanotubes (CNTs) can be considered as single or several layers of graphene sheet(s) rolling into a seamless cylindrical nanostructure. CNTs are further subdivided into single-walled CNTs (SWCNTs) and multi-walled CNTs (MWCNTs). SWCNTs have a better-defined diameter between 0.4 and 2 nm, whereas the outer diameter of MWCNTs ranges from 2 to 100 nm [55, 56]. SWCNTs and MWCNTs are up to a few microns in length, resulting in an extremely high aspect ratio (L/D) and large interfacial area. Due to their unique structure and nanotopography, CNTs exhibit great mechanical strength and chemical stability, as well as exceptional electrochemical and thermal properties [57]. These excellent properties have led to the wide application of CNTs in a variety of fields, including electrochemical devices [58], field emission devices [59], energy storage [60], probes [61], and medical applications [62].

**Fig. 1:**
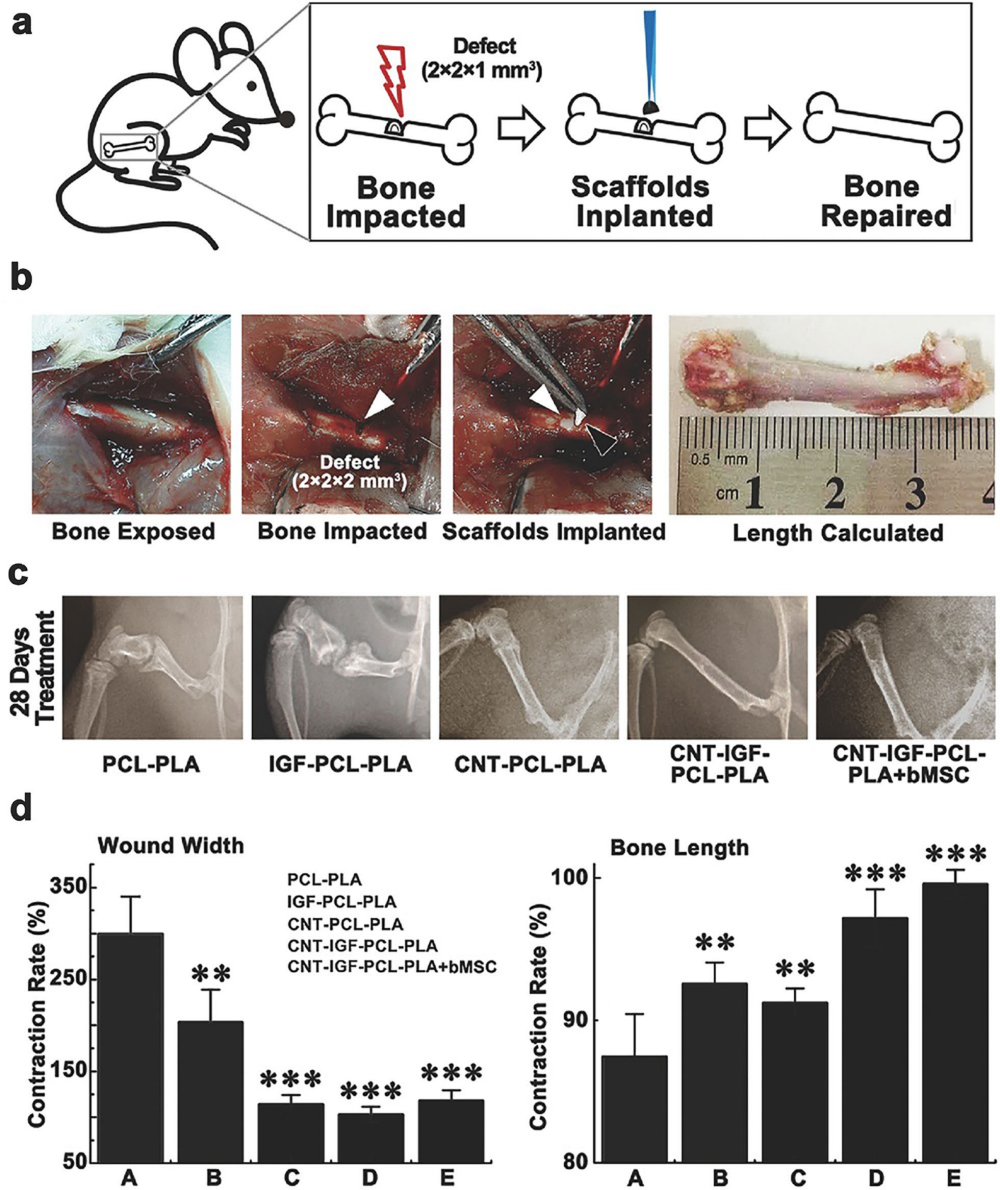
The osteogenic efficacy of IGF-doped CNT-coated PCL-PLA scaffolds. **a** Schematic illustration of the rat femoral defect model and scaffold implantation. **b** Exhibition of the operating procedure and scaffold implantation (white arrows, bone defects; black arrow, the implanted scaffold). **c** Representative radiographs of femurs 28 days after the operation. **d** Quantitative results of wound width and bone length. (Symbols: *, statistical significance with p < 0.05; **, statistical significance with 0.001 < p < 0.01; ***, statistical significance with p < 0.001 compared with the PCL-PLA group. The bars stand for standard deviations (n = 5).) [Panels **a**–**d** are from Chen et al. [85], reprinted with permission. Copyright © 2016 John Wiley and Sons]

Numerous studies have shown that CNTs alone could act as cores for initiating the crystallization of apatite/HA and accelerate the process of mineralization [63, 64]. High cell viability, strong adhesiveness, and an elevated proliferation rate were observed when osteoblastic cells were cultured in CNT-coated dishes [65]. It was indicated that the unique rough nanostructure of CNTs increased the surface roughness of the CNT-coated dishes, which up-regulated the expression of vinculin and resulted in better cell adhesion and viability. In another in vivo study [66], CNTs facilitated the process of bone formation and were eventually incorporated into the newly formed osseous tissue without causing any evident rejection reaction or inflammatory response, which corroborated the exceptional bone-tissue compatibility of CNTs. Notably, CNTs have been widely utilized as reinforcing agents for improving the structural integrity and mechanical properties of the biomaterials, including hardness, elastic modulus, tensile strength, bending strength, and compressive strength, as well as reducing the wear debris from composites [67–75]. In regard to tricomponent composite biomaterials, CNTs could serve as interfaces to increase the interfacial bonding between the other components [e.g., HA, bioactive glass (BG), polysaccharides, polymers, and mineral trioxide aggregate (MTA)] [75–79]. More importantly, the addition of CNTs into biomaterials (e.g., HA [68–70, 73, 80, 81] and polymers [67, 72, 74, 82–85]) substantially facilitated cell adhesion, proliferation, migration, mineralization, osteoinduction, and bone regeneration. For example, CNT-coated polycaprolactone-polylactic acid scaffolds combined with insulin-like growth factor-1 triggered osteogenic differentiation of MSCs, inhibited cellular senescence, and accelerated bone regeneration in a rat femoral defect model (Fig. 1) [85]. Moreover, due to the electrical conductivity of CNTs, the poly-dl-lactide (PLA)/CNTs composite scaffold exhibited favorable effects on osteoblast proliferation, extracellular mineralization, and osteogenic differentiation under electrical stimulation [86, 87].

Despite numerous exceptional properties, one factor that might hamper the application of CNTs is the underlying cytotoxicity. It has been reported that CNTs dispersed in solution could impair MSC proliferation, osteogenic differentiation, mineralization, and adipogenic differentiation via the Smad-dependent BMP signaling pathway [88]. As indicated above, pristine CNTs dispersed in the solution could elicit cytotoxicity and hinder cell activities, presumably by the production of ROS and activation of inflammatory reactions. However, CNTs bound to a surface exhibit minimal cytotoxicity, which suggests that surface functionalization might be critical to eliminating the potential cytotoxicity of CNTs. For example, PEGylated SWCNTs exhibit reduced ROS-mediated cytotoxic potency compared with uncoated SWCNTs [89]. In general, a more profound understanding of the cytotoxicity of CNTs is needed such that an optimal surface functionalization strategy can be designed to mitigate the hazard that might be associated with the application of CNTs.

In general, one-dimensional biomaterials (e.g., nanowires and nanotubes) exhibit unique nanotopography and a large length-to-diameter ratio, which exerts a great influence on cell behavior and calcium biomineralization. The diameter and orientation of aligned nanowires/nanotubes potentially represent the key parameters for MSC differentiation, which surely deserves more investigation. Moreover, the linear morphology of nanowires and nanotubes could be utilized to guide neovascularization and innervation, which may represent a hotspot of future research. Selective examples of one-dimensional biomaterials for bone tissue engineering are briefly presented in Table 2.

**Table 2:** Selective examples of one-dimensional biomaterials for bone tissue engineering

Biomaterial	Modification	Composite biomaterial	Fabricated into 3D scaffold?	In vitro model	In vivo model	Effect(s)	Ref.
SiNW	–	–	–	mMSCs	–	To support cell adhesion and proliferation; to promote osteogenic differentiation and chondrogenic differentiation	[45, 46]
BG-NFs	–	–	–	rMSCs	–	To support cell adhesion and proliferation; to promote osteogenic differentiation	[47]
PLGA/nHA-NFs	–	–	–	hMSCs	–	To support cell adhesion and proliferation; to promote osteogenic differentiation and calcium mineralization	[48]
TiO_2_-NTs	–	–	–	rMSCs	–	To promote cell adhesion, proliferation, migration, osteogenic differentiation, and calcium mineralization	[50]
TiO_2_-NTs	–	–	–	hMSCs	–	To support cell adhesion and proliferation; to promote osteogenic differentiation	[52]
TiO_2_-NTs	–	Titanium cylinder implants	–	–	Minipig skull drilling model	To accelerate osseointegration and bone remodeling in vivo	[54]
CNTs	Phosphonates-functionalized; PABS-functionalized	–	–	–	–	To promote calcium mineralization	[63]
CNTs	–	–	–	–	–	To promote calcium mineralization	[64]
CNTs	–	–	–	Saos-2	–	To promote cell adhesion and proliferation	[65]
CNTs	–	–	–	–	Mouse skull subperiosteal pocket model; mouse tibial bone defect model; mouse ectopic bone formation model using dorsal muscle pouch	To facilitate bone formation in vivo with great biocompatibility; to promote calcium mineralization	[66]
CNTs	Carboxylation	HA	–	MC3T3-E1	–	To improve mechanical properties; to promote cell adhesion, proliferation, and osteogenic differentiation	[68]
CNTs	Hydroxylation; carboxylation	[PCL/PLA]	Yes	rMSCs	Rat femoral bone defect model	To promote cell adhesion, proliferation, and osteogenic differentiation; to decelerate cellular senescence; to accelerate bone regeneration in vivo with great biocompatibility	[85]

*SiNW* vertically aligned silicon nanowire; *mMSCs* mouse mesenchymal stem cells; *BG-NFs* bioactive glass nanofibers; *rMSCs* rat mesenchymal stem cells; *PLGA/HA-NFs* nanofibers comprised of poly(lactide‐co‐glycolide) and nano‐hydroxyapatite; *hMSCs* human mesenchymal stem cells; *TiO2-NTs* titanium dioxide nanotubes; *CNTs* carbon nanotubes; *PABS* poly(aminobenzene sulfonic acid); *Saos-2* human osteosarcoma cells; *HA* hydroxyapatite; *MC3T3-E1* mouse preosteoblastic cells; *PCL* polycaprolactone; *PLA* polylactic acid; *Ref.* references

## Two-dimensional biomaterials

The definition of a two-dimensional biomaterial arises from the fact that only one dimension of it is within the nanoscale range (< 100 nm) [4]. Based on the definition, two-dimensional biomaterials are characterized by a high diameter-to-thickness ratio, resulting in exceptional properties such as great absorption capacity.

### Graphene and its derivatives

Graphene, a monolayer of dense honeycomb lattice formed by carbon atoms, is considered the building block for other carbon allotropes (e.g., fullerenes and CNTs) [9]. Since the experimental discovery of graphene in 2004 [90], graphene and its derivatives, including graphene oxide (GO) and reduced graphene oxide (rGO), have received tremendous attention for their potential application in tissue engineering [91].

**Fig. 2:**
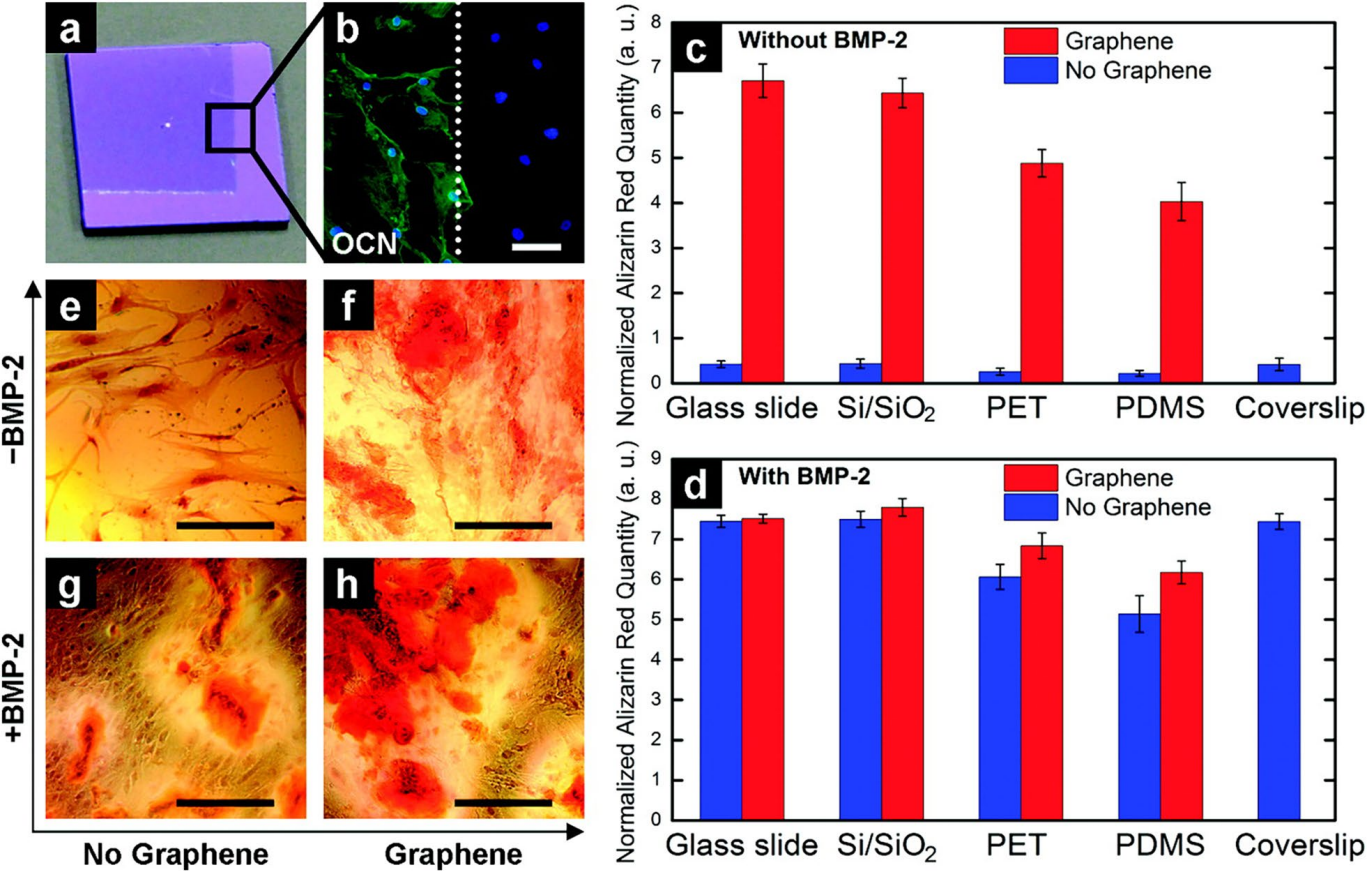
Graphene promotes hMSC osteogenic differentiation. **a** Photographs of a partially graphene-coated Si/SiO_2_ substrate (1 cm × 1 cm). **b** Osteocalcin (OCN) immunostaining of hMSCs showing the osteoinductive capability of graphene (white dotted line indicates the graphene boundary). **c**, **d** Quantification analysis of alizarin red S staining of hMSCs grown on different substrates with/without graphene for 15 days. **c** Cells grown without the presence of BMP-2 (compared with coverslips). **d** Cells grown with the presence of BMP-2 (compared with coverslips). **e–h** Alizarin red S staining of hMSCs cultured in different conditions reveals different amounts of calcium nodules. (Scale bars, 100 μm) [Panels **a**–**h** are from Nayak et al. [97], reprinted with permission. Copyright © 2011 American Chemical Society]

The peculiar hexagonal lattice nanostructure and one atomic thickness endow graphene with a large surface area, excellent mechanical properties, exceptionally high electronic conductivity, great thermal conductivity, superior charge carrier mobility, and impermeability to gases [91, 92]. Advances in the large-area synthesis of uniform graphene via chemical vapor deposition (CVD) have made it feasible to explore the potential biomedical applications of graphene [93]. Numerous studies have elucidated that CVD-grown graphene on different substrates (e.g., soda lime glass, polydimethylsiloxane (PDMS), polyethylene terephthalate (PET), oxidized silicon wafer (SiO_2_/Si stack), and stainless steel) exhibited favorable effects on cell adhesion [94, 95], proliferation [94, 95], and osteogenic differentiation of MSCs [96–98]. For instance, Nayak et al. [97] discovered that the osteogenic differentiation rate of MSCs co-cultured with graphene was comparable to that achieved by introducing the common growth factor BMP-2 (Fig. 2). It has also been reported that three-dimensional graphene foams exhibit great potential for maintaining MSC viability and inducing spontaneous osteogenic differentiation without extrinsic osteogenic inducers [98]. The exceptional osteoinductive property of graphene is attributed to its superior noncovalent binding ability, which allows graphene to serve as a preconcentration platform for osteogenic inducers [96]. Specifically, π–π stacking among the aromatic rings endows graphene with an extremely high absorption capacity of dexamethasone and β-glycerophosphate, which promote MSC differentiation toward the osteogenic lineage. Moreover, graphene was composited with other materials (e.g., BG and HA) as hybrid biomaterials [99–101]. The addition of graphene not only substantially enhanced the mechanical properties of these hybrid scaffolds (e.g., compressive strength and tensile strength), but also showed favorable biocompatibility and osteoinductivity in both in vitro and in vivo experiments.

Graphene oxide (GO) generally refers to the oxidized form of graphene, which incorporates diverse oxygen-containing functional groups (e.g., hydroxyl and epoxide groups on the basal plane, and carboxyl groups on the edge) within a single-atom-thick nanostructure [96, 102]. In addition to the active reaction sites provided by a variety of oxygen-containing groups, the regular distribution of these functional groups provides GO with both hydrophilic and hydrophobic parts along with the remaining carbon–carbon sp^2^ domains, all of which enable GO to have more active interactions with biomolecules via covalent, noncovalent, electrostatic, and hydrogen bonding. It has been reported that GO and surface-modified GO are capable of enhancing cell adhesion and proliferation, as well as facilitating HA mineralization and osteogenic differentiation [96, 103–107]. As mentioned above, the distinct structure endows GO with exceptionally high absorption capacity for various molecules, which explains its substantial influence on cell differentiation. According to Lee et al. [96], GO could concentrate dexamethasone via the remaining π–π stacking among the aromatic rings, and absorb ascorbic acid via hydrogen bonding, thus accelerating osteogenic differentiation of MSCs. In addition, the hydrogen bonding and electrostatic interactions between GO and insulin help with the focal enrichment of insulin while preserving the insulin protein structure, thus promoting adipogenic differentiation of MSCs in adipogenic medium [96]. However, it has also been suggested that the physical stress derived from the topographic features of GO may act as another essential parameter in promoting osteogenic differentiation, presumably by affecting cytoskeletal tension and inducing cytoskeletal reorganization [103]. The incorporation of GO with other biomaterials has been the focus of intensive research in the past few years. As might be expected, even a tiny amount of GO (e.g., 1 wt%) could boost the mechanical strength of the composites to a large extent [108–122], whereas excess GO may jeopardize the mechanical performance presumably by forming agglomerates and increasing porosity [108, 114, 115, 121]. The hydrophilicity and water retention ability of the composite scaffolds are also improved by the incorporation of GO [110, 111, 114, 117, 118, 122, 123], which partially contributes to the improved cell adhesion strength and molecule absorption affinity of the composites. During the fabrication of some tricomponent or tetracomponent composite biomaterials, GO also serves as an interface phase to facilitate the interfacial binging of the other components (e.g., polymers and bioceramics) [111, 112, 116, 118, 120, 122]. Most importantly, the GO-incorporated hybrid scaffolds exhibited great biocompatibility [108–113, 116–118, 120–129], antibacterial activity [110, 127], and osteoinductivity [109, 111, 112, 114, 116, 117, 123, 125–128, 130] both in vitro and in vivo, which was corroborated by elevated ALP activity, calcium mineralization, and osteogenic gene expression. For instance, Zhang et al. [128] designed a novel bifunctional bioceramic scaffold by incorporating Fe_3_O_4_ nanoparticles into GO-modified β-tricalcium phosphate (β-TCP) scaffold. As magnetic particles, Fe_3_O_4_ endowed the composite scaffolds with excellent magnetothermal effects under a magnetic field, which could be precisely controlled by altering the magnetic field intensity and the content of Fe_3_O_4_ nanoparticles. In vitro experiments demonstrated that the prominent magnetothermal effect of the composite scaffolds reduced the cell viability of osteosarcoma cells (MG-63) by 75%, whereas GO and continuously released Fe^3+^ ions could synergistically accelerate osteogenic differentiation of MSCs and facilitate new bone formation. Of note, various bioactive molecules (e.g., osteogenic inducers [113, 122, 129, 131] and antibacterial nanoparticles [113]) have been immobilized into many GO-incorporated hybrid scaffolds to further improve the performance of the composite scaffolds. Given its active interactions with biomolecules, GO served as an effective carrier in these drug-loading scaffolds to achieve large loading dose and sustained release of the bioactive molecules, with preservation of the bioactivity of the molecules.

As implied by the name, reduced graphene oxide (rGO) is basically produced by reducing GO via different reduction techniques, such as chemical reduction and thermal reduction. During the reduction process, the quantities of oxygen-containing functional groups would be reduced to varying extents depending on the reduction methods, which results in modulated electrical conductivity, thermal stability, and hydrophilicity [132, 133]. Numerous studies have reported that rGO exhibited a favorable capability of supporting cell proliferation and adhesion, as well as promoting biomimetic mineralization and osteogenic differentiation [103, 134–137]. Inspired by its excellent capability of augmenting osteogenesis, rGO has also been incorporated with many other biomaterials (e.g., HA and polymer) to fabricate novel hybrid materials with superior biocompatibility and osteoinductivity [138–141].

Similar to any other carbon-based material, the cytotoxicity of graphene and its derivatives has always been a topic of great interest. Pristine graphene dispersed in the solution was reported to accumulate on the cell membrane and cause cytotoxicity, presumably by increasing intracellular oxidative stress and inducing apoptosis [142]. High concentrations of GO in the solution could also attenuate cell viability in a dose-dependent manner [143, 144]. However, graphene films coated onto different substrates exhibited superior biocompatibility without any appreciable cytotoxicity [95]. In addition, no acute or chronic toxicity was observed after mice were intravenously injected with PEGylated graphene at a dose of 20 mg/kg, which was corroborated by hematological and histological analyses [145]. Based on the discussion above, we may arrive at the conclusion that surface functionalization or immobilization of graphene and its derivatives might mitigate or even eliminate the potential cytotoxic effect. Moreover, the size and dose of graphene and its derivatives might also represent essential parameters to influence their biocompatibility and cytotoxicity.

### Other thin film coatings on biomaterials

Aside from graphene and its derivatives, a great number of two-dimensional nanofilm coatings have been applied to traditional biomaterials to facilitate more intense integration of biomaterials and the biological environment, and thus to expedite the process of bone regeneration.

After clean glass surfaces were modified with amino (–NH_2_), MSCs cultured on these modified surfaces could spontaneously differentiate toward the osteogenic lineage even without the presence of extrinsic osteogenic inducers [146]. In another study, calcium phosphate coatings on substrates exerted a potent effect on triggering the spontaneous osteogenic differentiation of human MSCs without osteogenic additives, presumably by modulating the behavior of focal adhesions [147]. It has also been reported that TiO_2_-HA nanocomposite coatings favor MSC proliferation, adhesion, osteogenic differentiation, and extracellular matrix mineralization [148].

**Fig. 3:**
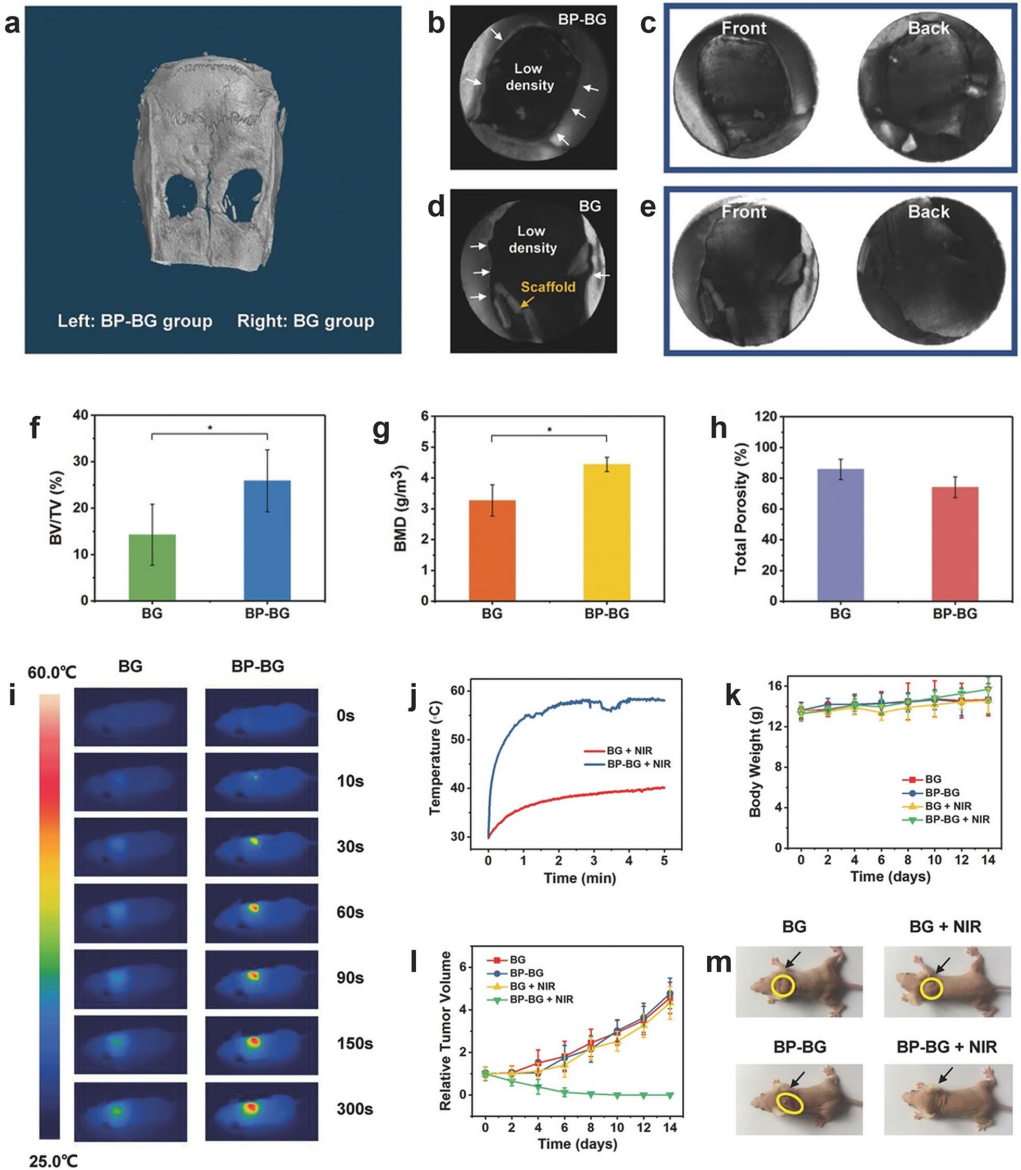
In vivo bone regeneration effect and photothermal effect of BP-BG scaffolds. **a–h** Micro-CT images and quantitative analysis of the harvested crania from SD rats after 8 weeks of implantation. **a** Micro-CT 3D reconstructed imaging of the harvested cranium. Micro-CT images of the BP-BG group (**b**, **c**) and BG group (**d**, **e**) were obtained by contrasting with black (**b**, **d**) and white (**c**, **e**) substrates, respectively, to visualize the newly formed osseous tissue. **f–h** Quantitative parameters indicating the bone regeneration effect of BG and BP-BG scaffolds, including bone volume/tissue volume (**f**), bone mineral density (**g**), and total porosity (**h**). **i–m** Photothermal tumor ablation induced by BP-BG scaffolds under NIR irradiation. **i** Infrared thermographic images of the tumor-bearing nude mice in different groups. Mice were implanted with BG or BP-BG scaffolds and subsequently treated with NIR irradiation for varied time intervals. **j** Real-time temperature in tumor sites corresponding to (**i**). **k** The average body weight of nude mice in different groups. **l** The average tumor volume of nude mice in different groups. The results demonstrated that the photothermal effect of BP-BG scaffolds was efficient in suppressing tumors. **m** Gross view of osteosarcoma-bearing nude mice in different groups on the 14th day. (Symbols: *, statistical significance with p < 0.05. Data is presented as mean ± SD.) [Panels **a**–**m** are from Yang et al. [149], reprinted with permission. Copyright © 2018 John Wiley and Sons]

Black phosphorus (BP) nanofilms have emerged as promising bioactive coatings for numerous applications due to their high photothermal conversion efficiency and fine osteoinductivity. Yang et al. [149] constructed a bifunctional therapeutic platform by incorporating BP nanofilm into the 3D-printed BG scaffold. The presence of BP nanofilms endowed the hybrid scaffold with excellent photothermal performance for the ablation of osteosarcoma, as well as escalated osteoconduction and osteoinduction performance to facilitate subsequent bone regeneration, which was corroborated both in vitro and in vivo (Fig. 3). One way to explain the exceptional bone regeneration performance of BP nanosheets is that exposure to the oxygen and water could lead to the rapid biodegradation of BP and result in the release of abundant ${\mathrm{PO}}_{4}^{3-}$, which rapidly extracts ${\mathrm{Ca}}^{2+}$ ions and accelerates the formation of calcium phosphate to facilitate the process of biomineralization and bone regeneration.

In general, two-dimensional nanosheets and nanofilms usually serve as coatings and layer-by-layer building blocks for the modification and fabrication of composite scaffolds. The large diameter-to-thickness ratio of two-dimensional nanofilm coatings endows them with great potential for altering the intricate surface properties of biomaterials, such as surface chemistry and charge, which subsequently influence cell behavior and cell fate in a profound way. Selective examples of two-dimensional biomaterials for bone tissue engineering are briefly summarized in Table 3.

**Table 3:** Selective examples of two-dimensional biomaterials for bone tissue engineering

Biomaterial	Modification	Composite biomaterial	Fabricated into 3D scaffold?	In vitro model	In vivo model	Effect(s)	Ref.
Graphene	–	–	–	hMSCs; Saos-2	–	To promote cell adhesion and proliferation	[94]
Graphene	–	–	–	hMSCs	–	To support cell adhesion and proliferation; to promote osteogenic differentiation and calcium mineralization	[97]
Graphene	Nitrogen-doped	HA + agarose	Yes	rMSCs	Rabbit femoral bone defect model	To improve mechanical properties; to promote cell adhesion, proliferation, osteogenic differentiation, and calcium mineralization; to accelerate bone regeneration in vivo	[101]
GO	–	–	–	cMSCs	–	To support cell adhesion and proliferation; to promote osteogenic differentiation and calcium mineralization	[106]
GO	–	β-TCP	Yes	hMSCs	Rabbit cranial defect model	To promote cell proliferation, osteogenic differentiation, calcium mineralization; to accelerate bon regeneration in vivo	[126]
rGO	–	HA	Yes	MC3T3-E1	Rabbit cranial defect model	To support cell adhesion and proliferation; to promote osteogenic differentiation and calcium mineralization; to accelerate bone regeneration in vivo	[141]
TiO_2_-HA nanocomposite coating	–	–	–	hMSCs	–	To promote cell adhesion, proliferation, osteogenic differentiation, and calcium mineralization	[148]
BP	–	BG	Yes	hMSCs; Saos-2	Rat cranial defect model; mouse subcutaneous tumor-bearing model	To support MSCs adhesion and proliferation; to promote MSCs osteogenic differentiation and calcium mineralization; to accelerate bone regeneration in vivo; photothermal effect in vitro and in vivo (for effective tumor ablation)	[149]

*hMSCs* human mesenchymal stem cells; *Saos-2* human osteosarcoma cells; *HA* hydroxyapatite; *rMSCs* rat mesenchymal stem cells; *GO* graphene oxide; *cMSCs* caprine mesenchymal stem cells; *β-TCP* β-tricalcium phosphate; *rGO* reduced graphene oxide; *MC3T3-E1* mouse preosteoblastic cells; *TiO*_*2*_ titanium dioxide; *BP* black phosphorus; *BG* bioactive glass; *Ref.*, references

## Three-dimensional biomaterials

Biomaterials with all dimensions larger than the nanoscale are defined as three-dimensional biomaterials, and most of the clinically used implants fall into this category. With tunable spatial structure and biochemical properties, three-dimensional biomaterials could act as imitated extracellular matrices to regulate cell behavior. It is worth mentioning that zero-, one-, and two-dimensional biomaterials are usually incorporated into three-dimensional scaffolds to combine their exceptional biological effects. Metallic scaffolds, bioceramic scaffolds, polymer scaffolds, and hydrogels are the most investigated three-dimensional biomaterials for bone regeneration.

### Metal-based scaffolds

Due to their great biocompatibility and superior mechanical strength, metal alloys (e.g., stainless steel, titanium alloys, and cobalt–chromium) have been widely utilized as plates for internal fixation of fractures and as protheses for joint replacement. Given the poor biodegradability of metal alloys, it would be difficult for newly formed osseous tissue to resorb and replace solid metal implants, which may hinder their application in bone tissue engineering. In fact, secondary surgery is usually required to remove the implanted metal plates after fracture healing is completed. On the other hand, metal ions and wear debris could be released from some metal implants via corrosion in the electrolytic body fluid, potentially resulting in local or systematic toxicity [150]. Of note, the elastic modulus of some metal implants is much higher than that of natural bone, which may elicit the stress shielding effect and subsequently result in osteopenia and even fracture recurrence [151]. To overcome these limitations, many porous metal-based scaffolds that resemble cancellous bone in microstructure have been fabricated via different methods (e.g., rapid prototyping, stack sintering, and 3D inkjet printing) [152–155]. In vitro and in vivo analyses revealed that the interconnected micropores and macropores of these scaffolds were favorable for cell proliferation, cell adhesion, cell migration, mineralization, and adsorption of proteins, indicating excellent osteoconductivity and osteoinductivity [152–155]. The highly porous structure allowed for cell infiltration, which made it possible for these metal-based scaffolds to be integrated into the newly formed bone [154]. Moreover, the tunable porosity makes it feasible to modify the mechanical strength of these scaffolds and thus to mitigate the stress shielding effect [154]. In addition to the aforementioned studies, considerable effort has been devoted to exploiting the surface modification of metal-base implants. For example, Vaithilingam et al. [156] functionalized Ti_6_Al_4_V titanium alloy with phosphonic acid self-assembled monolayers, which subsequently served as an interphase to immobilize biomolecules and drugs, such as paracetamol, onto the alloy. In another study, Gopi et al. [157] found that strontium-substituted HA/ZnO duplex-layer coatings on magnesium alloy significantly improved its corrosion resistance in simulated body fluid.

### Bioceramic scaffolds

Bioceramics are defined as ceramic biomaterials for biological applications. With favorable biocompatibility, surface reactivity, corrosion resistance, mechanical stiffness, and cost effectiveness, bioceramics have been widely utilized within clinical orthopedics, such as bioceramic coatings on joint replacement protheses and bioceramic granules/powders for filling osseous defects [158–160]. However, the brittleness and poor fatigue resistance of bioceramics, which could worsen with the increasing porosity, limit their utility as load-bearing scaffolds [158, 161]. Bioceramics are subdivided into bioactive bioceramics (e.g., calcium phosphate (CaP), bioactive glass (BG), calcium sulfate, and calcium silicate) and bioinert bioceramics (e.g., zirconia and alumina) based on whether chemical bonding could be formed between the bioceramics and living tissues after implantation [160]. Among these bioceramics, calcium phosphate (CaP) and bioactive glass (BG) are most commonly used for orthopedic and dental applications.

#### Calcium phosphate (CaP)

Calcium phosphates (CaPs) mainly include hydroxyapatite (HA), amorphous calcium phosphate (ACP), dicalcium phosphate (DCP), tricalcium phosphate (TCP), octacalcium phosphate (OCP), and biphasic calcium phosphates (BCPs) [159]. These CaPs can be manufactured in various forms, including powders, granules, coating layers, and bulk with tunable porosity and density. CaP-based biomaterials have the capability of integrating with the bone tissue without forming fibrous connective tissues or adipose tissues, indicating great bioactivity and osteoconductivity [162]. In fact, the chemical composition and structure of CaPs resemble those of native bon tissues, which also contribute to their excellent bioactivity and biocompatibility.

**Fig. 4:**
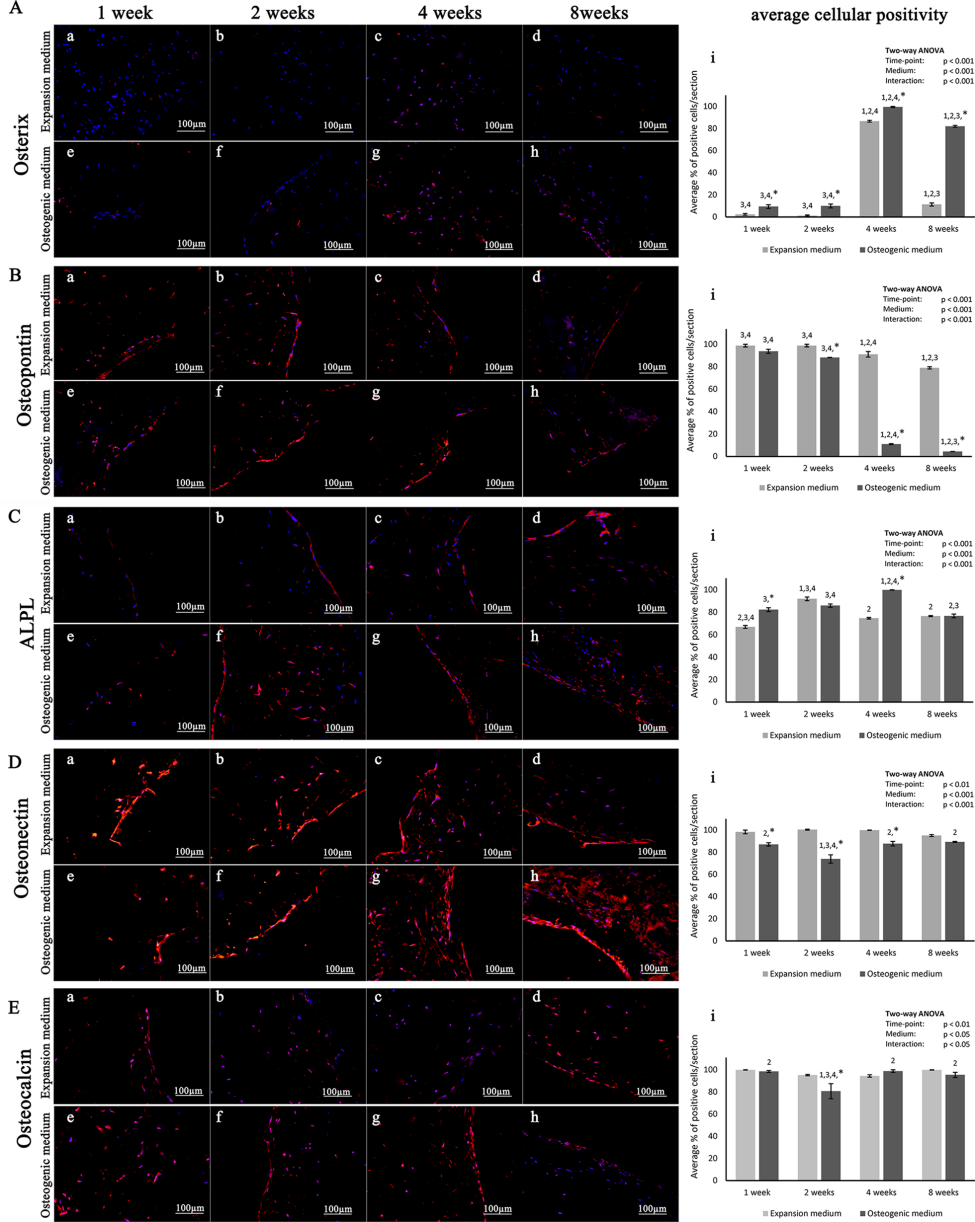
Collagen/Mg-doped HA scaffolds induce hADSCs osteogenic differentiation with/without extrinsic osteogenic inducers. **a**–**h** Representative immunohistochemical staining images of osteogenesis-related gene markers after hADSCs were cultured on scaffolds in different media for various time intervals. **i** Corresponding quantitative analysis of the percentage of positive cells for different osteogenic gene markers. (Symbols: *, significant difference when comparing osteogenic and expansion medium groups at the same time points; 1, significant difference compared with the 1-week group in the same medium; 2, significant difference compared with the 2-week group in the same medium; 3, significant difference compared with the 4-week group in the same medium; 4, significant difference compared with the 8-week group in the same medium) [Panels **A**–**E** are from Calabrese et al. [171]. Copyright © 2016 Calabrese et al.]

Hydroxyapatite (HA), which is typically denoted as Ca_10_(PO_4_)_6_(OH)_2_, is present in natural bone and teeth as an inorganic component of bone matrix. Up to 70% of the wet weight of human bone consists of HA, which intersperses in the collagen matrix as mosaics of microcrystallites [163]. As an intrinsic component of bone tissue, pure HA has drawn widespread attention for its application in bone tissue engineering. As a bioactive ceramic, HA forms strong chemical bonds with bone tissue, enabling active interactions with cells/biomolecules and regulation of cell fate. Aside from supporting cell adhesion and proliferation, HA-based scaffolds accelerate cell infiltration, expedite the process of mineralization, elevate ALP activity, upregulate the expression of osteogenic genes, and facilitate the process of angiogenesis, all of which indicate excellent biocompatibility, osseointegration, osteoconductivity, osteoinductivity, and angiogenic effects [164–171]. For example, Calabrese et al. [171] found that collagen/Mg-doped HA scaffolds could induce MSC differentiation toward the osteogenic lineage even in the absence of extrinsic osteogenic inducers (Fig. 4). Compared with other CaPs, HA has a relatively slow degradation rate, which may result in prolonged retention time in vivo (e.g., even up to several years). Given its brittleness and insufficient mechanical strength, HA minimally sustain mechanical stress during bone remodeling, which hinders its application in repairing large osseous defects [158, 161]. To address these limitations, biodegradable polymers have been incorporated with HA to improve the mechanical performance, reduce the brittleness, and modulate the biodegradability of composite scaffolds [164, 166, 169, 170, 172]. It is also worth mentioning that the addition of polymers into HA might endow the composite biomaterial with some additional beneficial properties, such as the capability to deliver biomolecules [166].

Tricalcium phosphate (Ca_3_(PO_4_)_2_), which is abbreviated as TCP, can be subdivided into several polymorphs (i.e., β-TCP, α-TCP, and α′-TCP) depending on the atomic arrangement [173, 174]. TCP has a calcium-to-phosphorus (Ca/P) ratio of 1.50, which is rather close to that of natural human bone tissue [175–177]. It has been reported that porous TCP scaffolds are highly bioactive, biocompatible, osteoconductive, and osteoinductive [178]. Compared with HA, TCP is much more resorbable and degradable, which could serve as an abundant source of calcium and phosphorus and thus facilitate the process of bone regeneration [175]. However, the brittleness, poor mechanical strength, excessive solubility, and high degradability of TCP hampered its application in load-bearing sites. A variety of polymers (e.g., alginate, polylactic acid (PLA), polycaprolactone (PCL), and collagen) were incorporated with TCP for modulating the biodegradation rate, as well as improving mechanical properties and osteoinductive performance of the composite scaffolds [179–182]. For instance, a novel type of injectable 3D scaffold with favorable mechanical strength and biological performance was fabricated by combining CaCl_2_-coated β-TCP beads with alginate hydrogels [179]. The instantaneous crosslinking between the alginate hydrogel and CaCl_2_ also endowed the composite with high injectability to form the custom-tailored shapes for osseous defects.

Another type of CaP that has been the focus of extensive research is biphasic calcium phosphate (BCP), which refers to an intimate mixture of two different CaPs. Generally, the most commonly used BCPs consist of HA and β-TCP in varying proportions [159]. As mentioned above, HA was relatively ‘stable’ with low biodegradability, whereas β-TCP undergoes biodegradation and dissolution at a much faster rate and results in an ionic-rich environment. The combination of HA and β-TCP would bring together the advantages of both, such as superior bioactivity, osteoconductivity, and osteoinductivity. Moreover, better control over the biodegradability and mechanical properties could also be achieved by altering the HA/β-TCP ratio of BCP, so as to yield a balance between bioactivity and mechanical stability.

In general, CaP-based biomaterials possess many advantages, such as superior biocompatibility, bioactivity, osteoconductivity, osteoinductivity, and cost effectiveness, all of which make them promising candidates for bone tissue engineering. On the other hand, more efforts are required to overcome the limitations of CaPs, namely brittleness, poor mechanical strength, presence of impurities, etc. More insights are also required to finely modulate the biodegradability and solubility of CaP-based biomaterials, which are determined by a variety of parameters (e.g., composition, microstructure, porosity, crystallinity, particle size range, and fabrication method).

#### Bioactive glass (BG)

Bioactive glass (BG) is considered a peculiar subgroup of ceramic biomaterials, typically with the composition of SiO_2_–Na_2_O–CaO–P_2_O_5_ [183, 184]. Since it was first fabricated by Professor Larry Hench in the late 1960s, BG has been extensively researched as one of the most promising biomaterials for tissue regeneration [184]. BG exhibits various clinical utilities, especially in the field of orthopedic and maxillofacial surgery, such as BG-based ossicular prostheses for the reconstruction of the ossicular chain [185] and BG-based bone substitutes for spinal fusion in the treatment of adolescent idiopathic scoliosis [186]. Generally, BG can be subdivided into three groups depending on the main component present in the composition, namely silicate (SiO_2_) glass, borate (B_2_O_3_) glass, and phosphate (P_2_O_5_) glass [187]. In addition, 45S5 Bioglass® (45 wt% SiO_2_, 24.5 wt% CaO, 24.5 wt% Na_2_O, 6 wt% P_2_O_5_) is the first and most famous BG originally synthesized by Hench and resembles human cancellous bone in terms of elemental composition and interconnected porous structure [184].

One of the biggest bright spots of BG is its fabulous surface reactivity and bioactivity, from which its name is derived. As elucidated by Hench [184, 188], the surface reaction of implanted BG could be summarized as follows: rapid release of soluble ions, formation of hydrated silica and hydroxy carbonate apatite bilayer on the surface, crystallization of HA, interaction and bonding with collagen fibrils produced by osteoblasts, enhanced adsorption and desorption of biomolecules, modulated cell behavior of macrophage and osteogenic cell line, all of which synthetically facilitate the process of mineralization and bone regeneration to a great extent. Incorporation of other oxides alters the performance of BG in various ways, such as the incorporation of AgO for the antibacterial effect and Al_2_O_3_ for strengthening the mechanical properties [187, 189]. As reported by several research groups, the addition of ZnO endowed BG with elevated bioactivity and mineralization rates, as well as favorable effects on the viability and differentiation of osteoblastic cell lines [190, 191]. Even a small variation in composition or a change in the proportion of each oxide could tremendously alter the physicochemical properties and biological performance of BG. It should also be emphasized that different manufacturing methods of BG, mainly including the melt-quenching route and sol–gel technique, may result in differences in uniformity, porous texture, dissolution rate, bioactivity, and mineralization ability [192, 193].

**Fig. 5:**
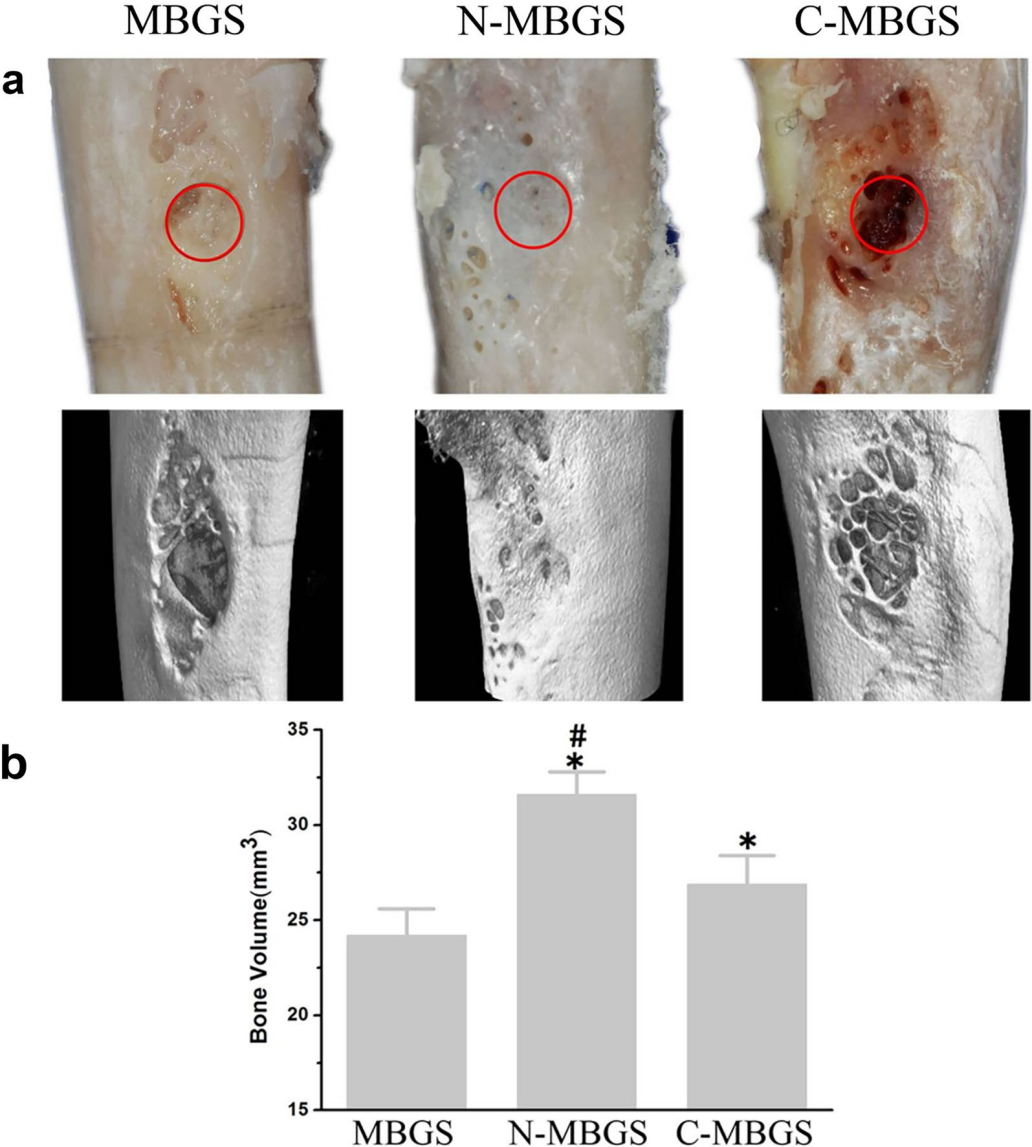
In vivo bone regeneration performance of MBGS, N-MBGS and C-MBGS in the rabbit femoral defect model after 12 weeks of implantation. **a** Photographs and the corresponding micro-CT 3D reconstructed images of rabbit femurs in different groups (red circles indicate the original sites of the bone defects). **b** Quantification of the newly formed bone volume after 8 weeks of implantation. (Symbols: *, significant difference compared with MBGS (p < 0.05); #, significant difference in comparison with C-MBGS (p < 0.05)) [Panels **a** and **b** are from Zhang et al. [196]. Copyright © 2016 Zhang et al.]

**Fig. 6:**
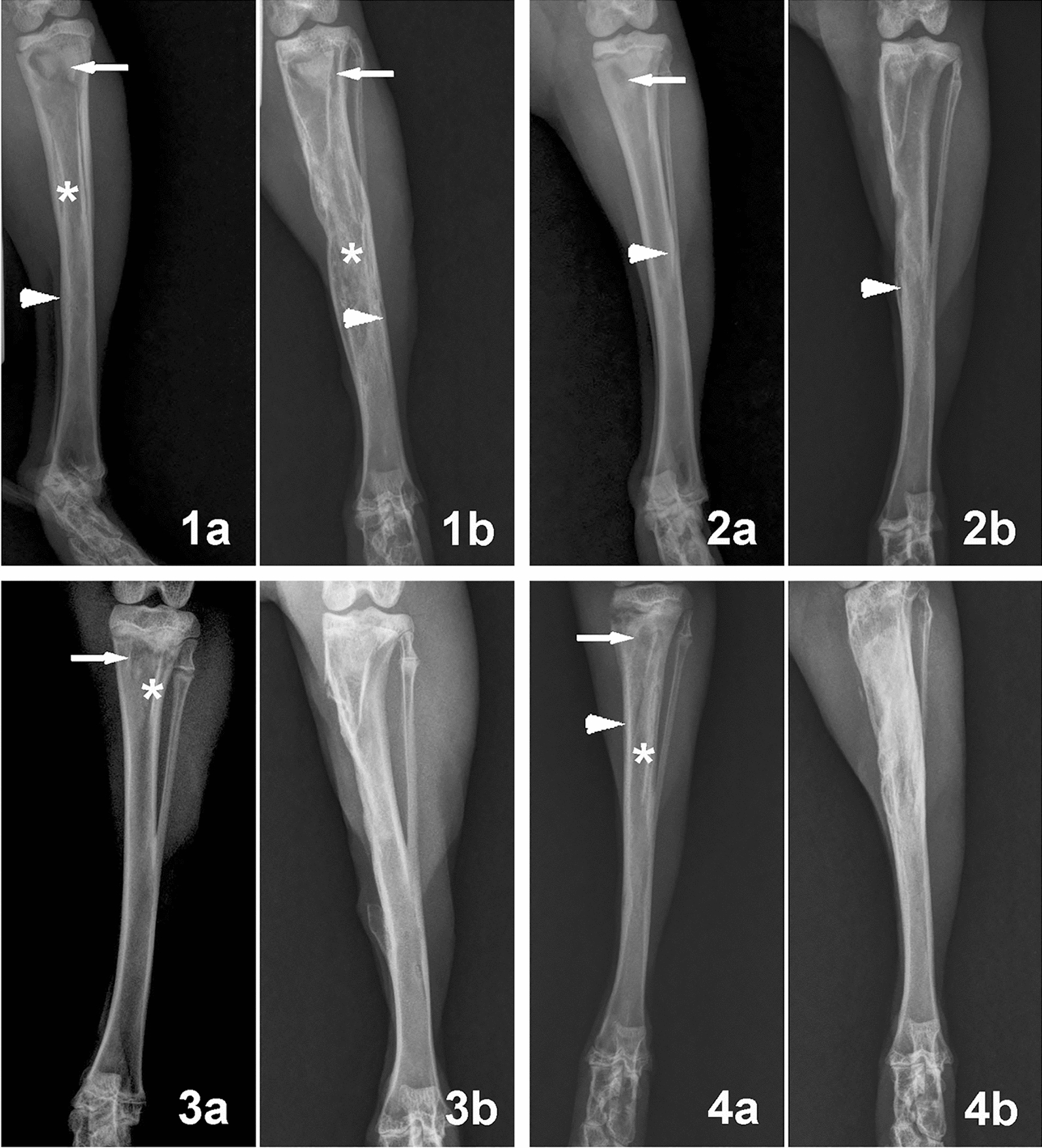
Representative radiographs of rabbit tibial osteomyelitis in different groups before surgery (**1a**, **2a**, **3a**, **4a**) and at 2 months after surgery (**1b**, **2b**, **3b**, **4b**). (Group 1, control group without any treatment; Group 2, debridement + daily intravenous administration of vancomycin for a month; Group 3, debridement + implantation of vancomycin-loaded calcium sulfate cement; Group 4, debridement + implantation of vancomycin-loaded borate BG cement.) In Group 1, the destroyed bone (arrows), periosteal newly formed osseous tissue (arrowhead), and sequestral bone formation (*) in the postoperative radiographs indicated the deterioration of osteomyelitis. The osteomyelitis in Group 2 was partly controlled, whereas osteomyelitis in Group 3 and Group 4 healed. [Panels **1a**–**4b** are from Ding et al. [199]. Copyright © 2014 Ding et al.]

Despite all the merits, such as superior bioactivity and osteoinductivity, the inherent brittleness of BG could result in fast porous structure collapse, presenting as a major obstacle in the context of bone tissue regeneration. It has been reported that polymer impregnation into BG allows better manipulation of mechanical properties and biodegradation rates, which subsequently guarantees the structural integrity and stability of composite biomaterials while facilitating bone regeneration [194, 195]. The combination of polymers with BG would bring together the advantages of both, facilitating improved bioactivity, mechanical properties, mineralization capability, osteoconductivity, and osteoinductivity. Another emerging biomaterial is mesoporous bioactive glass (MBG) with the pore size between 2 and 50 nm. Zhang et al. [196] fabricated MBG via a simple powder processing technique, yielding preferable compressive strength over MBG synthesized via the traditional polyurethane foam template method. They also discovered that amino-functionalized MBG scaffolds (N-MBGS) exerted more favorable effects on MSC adhesion, proliferation, and osteogenic differentiation than unfunctionalized MBGS and carboxylic-functionalized MBGS, which was further corroborated in the rabbit femoral defect model (Fig. 5). The improved bioactivity, biocompatibility, osteoconductivity, and osteoinductivity of N-MBGS was ascribed to its positively charged surface and decreased degradation rate. Due to the extraordinary drug loading capacity of MBG, a variety of drugs (e.g., ipriflavone and gentamicin sulfate) were immobilized onto MBG to achieve a large loading dose and controlled release, which could endow MBG with anti-osteoporotic and antibiotic abilities [197, 198]. To meet the demands for custom-tailored bone graft substitutes, injectable BG cement was designed by mixing borate BG particles and chitosan-based bonding solution, yielding excellent mechanical strength, biocompatibility, and osteoinductive effects both in vitro and in vivo [199–201]. Moreover, Ding et al. [199] incorporate vancomycin into injectable borate BG cement as a multifunctional platform for the treatment of osteomyelitis in the rabbit tibia (Fig. 6).

### Polymer scaffolds

Biocompatible polymers are considered promising candidates for bone tissue engineering due to their excellent biocompatibility and design flexibility. The degradability of a polymer scaffold is determined by various factors, such as the composition of the polymer and the porosity of the polymer scaffold. According to the origin, polymers can be roughly categorized into two groups, namely natural polymers and synthetic polymers [202].

Natural polymers utilized for bone tissue regeneration mainly include collagen, chitosan, hyaluronic acid, silk, alginate, etc. One potential advantage of natural polymers is that they may contain some biological recognition sites, which would help with the specific interaction with cells and therefore modulate cell behavior [203]. However, the potential presence of immunogen and pathogenic impurities, the poor cost effectiveness and unsatisfactory batch-to-batch replicability may hinder the application of natural polymers in bone tissue regeneration [202]. Moreover, the suboptimal processability of natural polymers proves to be an obstacle as well, which only allows limited control over the mechanical properties and degradation rates. A wide range of crosslinking techniques have been intensively explored to reinforce the mechanical strength of natural polymers, such as the use of chemical crosslinking reagents [204], enzymatic reaction of lysyl oxidase [205], and photocrosslinking method [206]. In addition to crosslinking, fiber orientation is another significant parameter for reinforcing the structural integrity of natural polymer-based scaffolds [207].

Synthetic polymers within the field of bone tissue engineering include polylactic acid (PLA), poly(glycolic acid) (PGA), poly(lactic-co-glycolic acid) (PLGA), poly(ethylene glycol) (PEG), polycaprolactone (PCL), etc. Synthetic polymers have received considerable attention in bone regeneration applications, which could be ascribed to their favorable effect on supporting cell attachment and propagation as well as their ability to promote MSC differentiation toward the osteogenic lineages and accelerate calcium biomineralization [208–211]. Compared with natural polymers, synthetic polymers are replicable and could be more easily tailored in terms of the microstructure, hydrophilicity, pore size, porosity, mechanical characteristics, and degradability [208–213]. As indicated in many studies, pore size and porosity are two of the key parameters for modulating the mechanical properties of synthetic polymer-based scaffolds [210–213]. Advanced fabrication techniques such as rapid prototyping and electrospinning were applied to construct the interconnected porous microstructure of polymer-based scaffolds with great precision [165, 214, 215]. Furthermore, a variety of soluble particles, known as porogens, could be imbedded into polymers to form porous structures upon dissolution, of which the pore size and porosity can be altered by changing the size and amount of porogens [210, 211, 213, 216, 217]. However, some synthetic polymers exhibit unsatisfactory hydrophilicity without secondary modification, which may hinder cell attachment and lead to poor biological performance [209]. Moreover, although various synthetic polymers are biocompatible and biodegradable, the release of degradation byproducts and wear debris might still elicit inflammatory responses and rejection reactions [218].

**Fig. 7:**
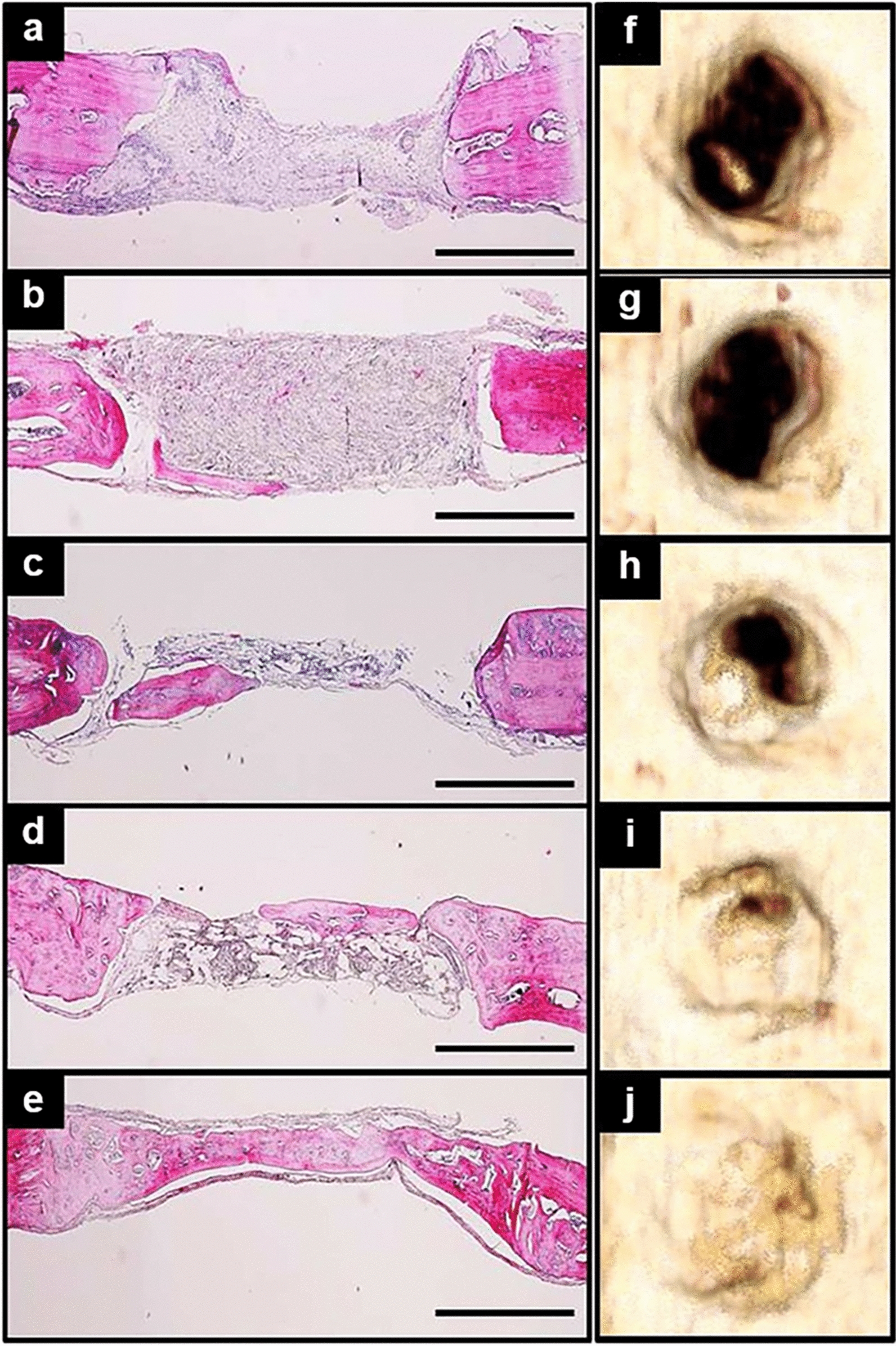
In vivo bone regeneration performance of different polymer scaffolds. **a**–**e** H&E staining of harvested rat crania in different groups after 4 weeks of implantation (**a** control; **b** silk scaffold; **c** PLGA scaffold; **d** PLGA–silk scaffold; **e** PLGA–silk–HA scaffold). (scale bar, 1 mm). **f**–**j** Corresponding micro-CT 3D reconstructed images of crania in different groups (**f** control; **g** silk scaffold; **h** PLGA scaffold; **i** PLGA–silk scaffold; **j** PLGA–silk–HA scaffold). [Panels **a–j** are from Sheikh et al. [221], reprinted with permission. Copyright © 2015 John Wiley and Sons]

**Fig. 8:**
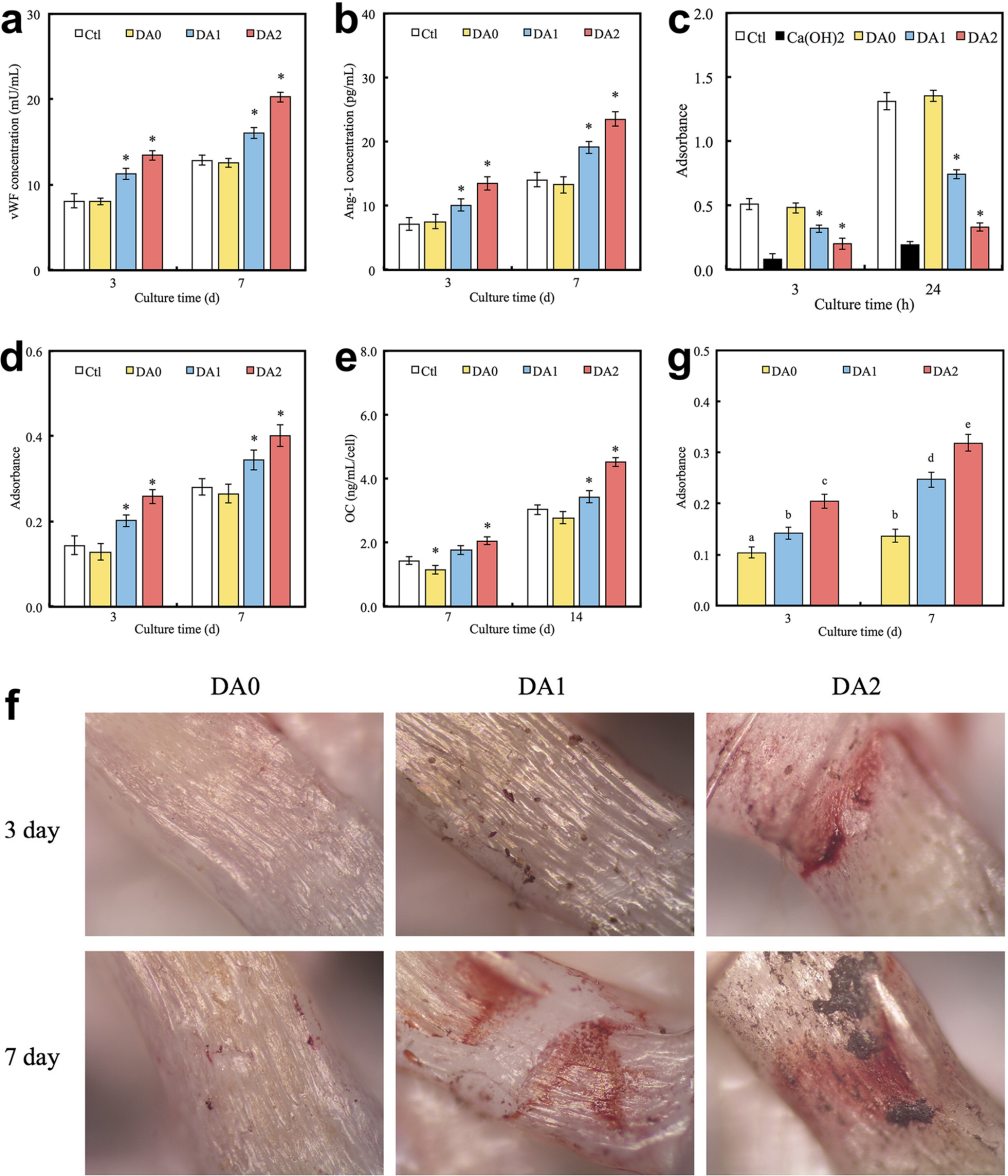
Multifunctionality of PDA/PLA scaffolds. **a**, **b** Protein expression levels of angiogenic markers (vWF and Ang-1) in hADSCs cultured on different substrates for various time intervals (Symbols: *, statistical significance (p < 0.05) compared with DA0). **c** Antibacterial performance of the PDA/PLA scaffold, evaluated by culturing Staphylococcus aureus on different substrates for 3 and 24 h (Symbols: *, statistical significance in comparison with DA0). **d**, **e** Osteogenic performance of the PDA/PLA scaffold, indicated by ALP activity (**d**) and osteocalcin secretion (**e**) from hADSCs cultured on different substrates for different time intervals (Symbols: *, statistical significance (p < 0.05) in comparison with DA0). **f**, **g** Alizarin red S staining of hDPCs cultured on different substrates for 3 and 7 days and the corresponding quantitative analysis of calcium mineralization. Values that do not share the common letter differ significantly from each other (statistical significance with p < 0.05). (*DA0* PLA scaffold without PDA coating; *DA1* PLA scaffold with 1 mg/ml PDA coating; *DA2* PLA scaffold with 2 mg/ml PDA coating; *Ctl* tissue culture plate was used as the control; *hDPCs* human dental pulp cells) [Panels **a**–**g** are from Kao et al. [226], reprinted with permission. Copyright © 2015 Elsevier]

Generally, both natural and synthetic polymer scaffolds with high porosity exhibit relatively inferior loading-bearing capacity compared with metallic biomaterials. To circumvent the possible mechanical failure of polymer-based scaffolds, calcium phosphate and bioactive glass were incorporated into the polymer matrix to fabricate composite scaffolds, resulting in improved biological performance and osteoinductive effects [165, 213, 214, 216, 219–224]. For instance, Sheikh et al. [221] demonstrated that incorporation of silk and HA nanoparticles into PLGA-based scaffolds could impart optimized stress-bearing capacity and hydrophilicity to the hybrid scaffolds along with better biocompatibility and bioactivity to facilitate cell growth and infiltration. In vivo results from rat cranial defect models showed that, when compared with the other scaffolds, silk-HA-PLGA composite scaffolds induced relatively more complete intramembranous ossification 4 weeks after implantation with no sign of inflammation or rejection (Fig. 7). Surface modification of polymer scaffolds with biomolecules, such as plasma deposition and Arg–Gly–Asp tripeptide (RGD), could substantially promote cell attachment and colonization [217, 225]. It is also worth noting that polydopamine (PDA) coating on the PLA scaffolds not only enhanced MSC adhesion, proliferation, and osteogenic differentiation, but also enabled the composite scaffolds to exhibit superior antibacterial effects and angiogenic effects (Fig. 8) [226].

### Hydrogels

Hydrogels are a group of hydrophilic polymeric materials that are able to absorb a vast amount of water and keep it retained, which could be ascribed to their crosslinked three-dimensional networks. Due to their hydrophilic nature and high water content, hydrogels have been considered promising candidates to mimic the natural hydrated microenvironment for cell growth. The crosslinked microstructure and adjustable architecture of hydrogels also guarantees their structural stability and integrity while being tailored into customized shapes. Moreover, hydrogels exhibit great permeability to nutrients, oxygen, metabolites, and other water-soluble bioactive molecules, making them ideal substrates for supporting cell growth. The degradability of hydrogels is dependent on a variety of factors, such as the composition of the hydrogels and the cross-linking density. It is also worth mentioning that many hydrogels could undergo the sol-to-gel phase transition in response to shifting environmental conditions (e.g., temperature), and the superior injectability of these hydrogels allows them to form custom-tailored shapes for repairing tissue defects [227, 228]. In recent decades, a variety of hydrogel-based biomaterials have been utilized within the field of tissue engineering to fabricate biomimetic tissues, such as skin and cartilage [229–231].

According to the polymer origin, hydrogels fall into three major categories: natural hydrogels, synthetic hydrogels, and hybrid hydrogels [232]. Natural hydrogels are composed of natural polymers (e.g., hyaluronic acid, alginate, fibrin, collagen, silk, gelatin, agarose, and chitosan), and possess numerous advantages such as low toxicity, high biocompatibility, inherent biodegradability, great bioactivity and cell affinity [229, 231, 233–236]. However, the applications of natural hydrogels in the field of bone tissue regeneration are quite limited by their relatively inferior mechanical properties, potential immunogenicity, and their unsatisfactory replicability and processability. To address these limitations, synthetic hydrogels (e.g., PEG-based hydrogels and PLA-based hydrogels) have emerged as alternative candidates for fabricating tissue substitutes [237, 238]. The excellent plasticity and reproducibility of synthetic hydrogels allows precise manipulation of the physiochemical properties of hydrogels during the process of polymerization and subsequent modification (e.g., crosslinking and functionalization), so as to custom-tailor the hydrogel constructs in terms of block structure, viscosity, mechanical properties, and biodegradability. Furthermore, natural/synthetic hybrid hydrogels were designed to bring together the advantages of both types of hydrogels, namely high cell/biomolecule affinity and bioactivity of natural hydrogels as well as better mechanical strength and processability of synthetic hydrogels [227, 228].

**Fig. 9:**
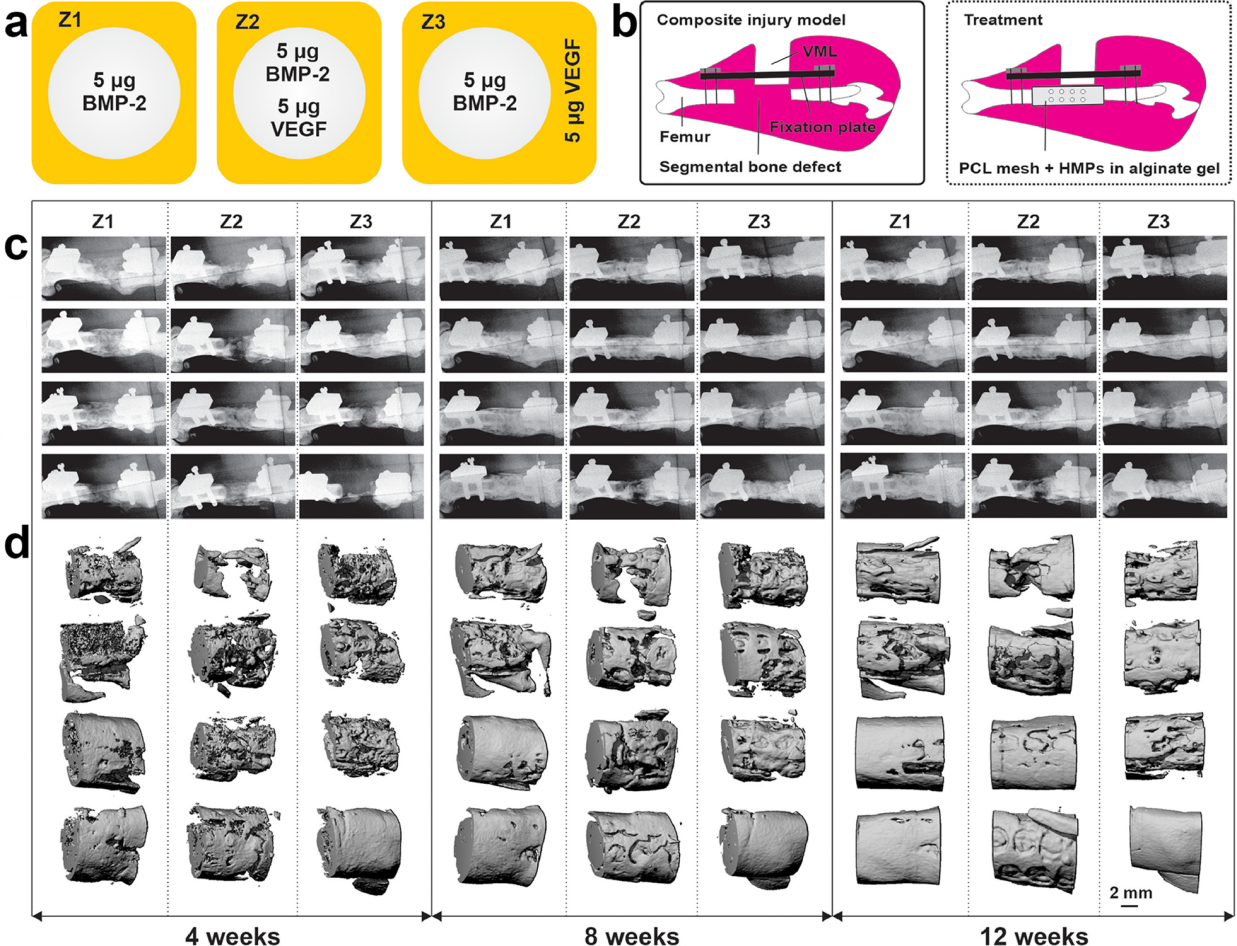
Quantitative evaluation of the bone regeneration effect of different release patterns in the rat femoral bone-muscle composite injury model. **a** Schematic illustration of the HMP-alginate hydrogel delivery systems. **b** Schematic illustration of the femoral bone-muscle composite injury model and treatment. **c** Longitudinal radiographs of the defect regions from different groups at the 4th, 8th, and 12th week post surgery. **d** Micro-CT 3D reconstructed images of the defect regions from different groups at the 4th, 8th, and 12th week post surgery. [Panels **a**–**d** are from Subbiah et al. [248], reprinted with permission. Copyright © 2020 Elsevier]

A great number of studies have demonstrated hydrogels’ favorable effect on supporting cell adherence and ingrowth as well as fostering intercellular interactions [227, 228, 230, 231, 234–236, 239–241]. With regard to bone tissue regeneration, both in vitro and in vivo studies have corroborated that hydrogel-based biomaterials facilitate the process of osteogenesis and calcium biomineralization substantially, and promote angiogenesis as well [236, 239–241]. Hydrogels incorporated with other types of biomaterials, such as calcium phosphates (CaPs) and demineralized bone matrix (DBM), exhibit reinforced mechanical strength and synergistic accelerating effects in bone regeneration [242, 243]. As indicated in many studies, incorporation or encapsulation of various bioactive biomolecules (e.g., RGD, heparin, BMP-2, fibronectin, fibrinogen, bisphosphonate, and growth factors) into hydrogels could potentiate cell adhesion and propagation as well as the osteogenic and angiogenic efficacy of the hybrid scaffolds [233, 236, 238, 243–248]. Notably, the incorporation of some biomolecules, such as heparin and RGD, could alter the mechanical properties of the hydrogels to a large extent [238, 246]. The heparin-functionalized hydrogel has emerged as a novel biomaterial for tissue regeneration and drug delivery given the excellent bioactivity of heparin [243–246, 248, 249]. The high electronegative charge of anionic heparin endows it with exceptional affinity to a variety of biomolecules (e.g., BMP-2, growth factors, fibronectin, chemokines, and antithrombin III), which substantially increases the loading dose of the biomolecules while facilitating sustained release at a desirable rate and preserving the biomolecules from denaturation. Subbiah et al. [248] developed an injectable delivery system by immobilizing BMP-2 and VEGF into a heparin-functionalized alginate hydrogel. The high loading efficiency and controlled release pattern of each growth factor were corroborated in vitro. Using this tunable dual growth factor delivery system, three diverse release patterns (i.e., mere BMP-2, simultaneous release of VEGF and BMP-2, sequential release of VEGF and BMP-2) were designed, and the osteogenic efficacy of each release pattern was examined in the rat femoral bone-muscle composite injury model. Evident bone regeneration was observed in all treatment groups, whereas the sequential release of VEGF and BMP-2 resulted in much more mineralized bone matrix and more developed vascular networks (Fig. 9).

**Fig. 10:**
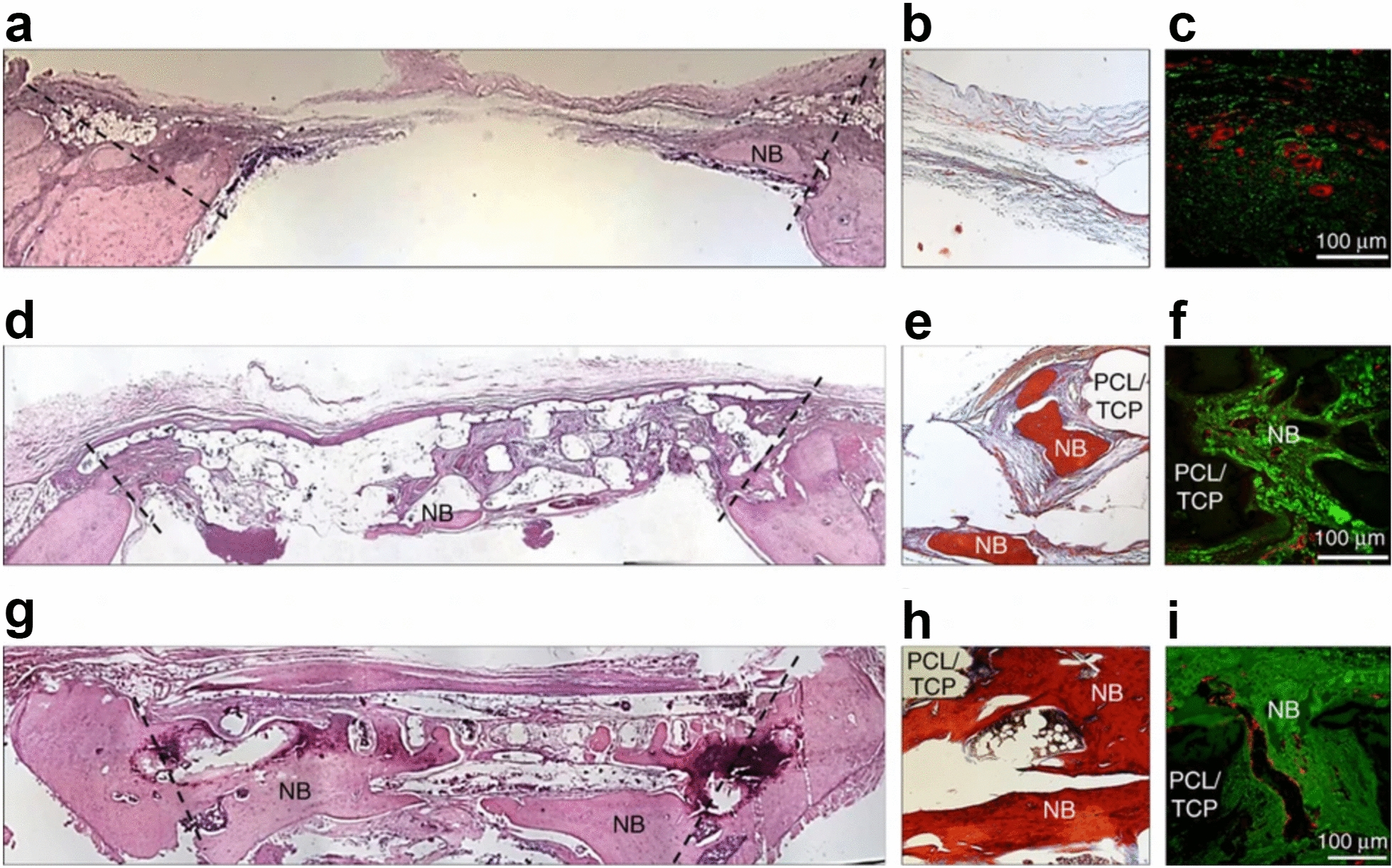
Qualitative evaluation of bone regeneration after implantation of bioprinted substitutes. **a**–**i** Histological and immunohistological staining of rat crania from different groups (**a**–**c**, untreated group; **d**–**f**, cell-free scaffold group; **g**–**i**, hAFSCs-laden substitute group) after 5 months of implantation. H&E staining images (**a**, **d**, **g**), modified tetrachrome staining images (**b**, **e**, **h**), and vWF immunostaining images (**c**, **f**, **i**). In tetrachrome staining images, red areas indicate mature bone, while blue areas indicate the lining of lacunae and osteoids. In vWF immunofluorescent images, red signals indicate blood vessels. (Symbols: NB, newly formed bone; PCL/TCP, the remaining scaffold) (*hAFSCs* human amniotic fluid-derived stem cells) [Panels **a**–**i** are from Kang et al. [239], reprinted with permission. Copyright © 2016 Springer Nature]

The growing attention given to hydrogels is partially ascribed to the increased use of 3D bioprinting in regenerative medicine fields. Technically speaking, 3D bioprinting is an emerging technology that utilizes cell-laden biocompatible materials, which are also known as bioinks, to design and manufacture living tissue-like structures in an additive layer-by-layer manner. This innovative technology makes it feasible to fabricate living constructs with predesigned structure and geometry, which also allows precise spatial manipulation of the cells and other components within the constructs. Bioinks are mostly composed of suspended cells in tandem with pregel extracellular matrix mimics, which typically contain nutrients and various biomolecules to maximally simulate natural extracellular environments. Given their excellent biocompatibility, plasticity, and similarity to natural extracellular matrix, hydrogels have been extensively explored as the main components of bioinks [239, 240, 250, 251]. For instance, Kang et al. [239] used hydrogel-based bioinks to fabricate rat cranial bone substitutes and examined their osteogenic capacity in a rat cranial bone defect model. Compared with the untreated defect group, more vascularized bone tissue was observed throughout the bioprinted substitutes at 5 months after implantation (Fig. 10).

In conclusion, three-dimensional materials are intensively utilized as the foundation frameworks for bone substitutes, which are usually combined with a variety of zero-, one-, two-, and other three-dimensional materials to deliver synergistic effects for bone regeneration. The interconnected porous microstructure of 3D scaffolds facilitates the process of osseointegration, neovascularization, and neurotization. 3D scaffolds with hierarchical porous structure (i.e., macropores and micropores) are the optimal choice for bone regeneration. Specifically, the macropores (with diameters of several hundreds of microns) could facilitate cell migration and neovascularization, whereas micropores (with diameters ranging from several microns to several tens of microns) favor the adsorption and retention of various bioactive biomolecules. Surface modification of three-dimensional materials profoundly improves their biological performance, whereas immobilization of diverse biomolecules (e.g., BMP-2, growth factors, and non-steroidal anti-inflammatory drugs) endows 3D scaffolds with more alluring properties. Furthermore, the advent of the 3D printing technique paves the way for the detailed design and precise fabrication of cell-laden biomimetic biomaterials, which will certainly be the focus within the field of bone tissue engineering. Selective examples of three-dimensional biomaterials for bone tissue engineering are briefly presented in Table 4.

**Table 4:** Selective examples of three-dimensional biomaterials for bone tissue engineering

Biomaterial	Modification	Composite biomaterial	Immobilization of biomolecules	Cell-laden?	In vitro model	In vivo model	Effect(s)	Ref.
Fe–Mg	–	–	–	–	MC3T3-E1	–	Similar to natural bone in terms of mechanical properties; desirable corrosion rate; to support cell adhesion, infiltration (into the porous scaffold), and proliferation	[154]
HA	–	Collagen	–	–	mMSCs	Mouse subcutaneous pocket model	To improve mechanical properties; to support cell adhesion, infiltration, and proliferation; to promote osteogenic differentiation; to induce angiogenesis and osteogenesis in vivo	[170]
HA	Mg-doped	Collagen	–	–	hMSCs	–	To support cell adhesion, infiltration, and proliferation; to promote osteogenic differentiation and calcium mineralization	[171]
β-TCP	–	Alginate	–	hMSCs	hMSCs	Mouse subcutaneous pocket model	To improve mechanical properties; to support osteogenic differentiation; to induce osteogenesis in vivo	[179]
MBG	Amination; carboxylation	–	–	–	rabMSCs	Rabbit femoral bone defect model	To improve mechanical properties; to support cell adhesion; to promote cell proliferation, and osteogenic differentiation; to improve bone regeneration in vivo	[196]
Borate BG	–	Chitosan	Vancomycin	–	MRSA	Rabbit MRSA-induced osteomyelitis model	To facilitate calcium mineralization; antibacterial effect; obvious therapeutic effect on osteomyelitis in vivo; to improve bone regeneration in vivo	[199]
PLA	PDA-coated	–	–	–	hMSCs	–	To promote cell adhesion, proliferation, angiogenic differentiation, osteogenic differentiation, and calcium mineralization; anti-bacterial effect	[226]
Gelatin-alginate hydrogel	–	–	–	hMSCs	hMSCs	Mouse subcutaneous pocket model	To support cell adhesion, proliferation, and osteogenic differentiation; to induce osteogenesis and angiogenesis in vivo	[240]
HMP-alginate hydrogel	–	–	BMP-2; VEGF	–	MVFs growth model	Rat femoral bone-muscle composite injury model	A delivery system with high loading efficiency and tunable release pattern; to improve microvascular network growth in vitro; to accelerate bone regeneration and vascularization in vivo	[248]

*Fe–Mg* iron–manganese alloy; *MC3T3-E1* mouse preosteoblastic cells; *HA* hydroxyapatite; *mMSCs* mouse mesenchymal stem cells; *hMSCs* human mesenchymal stem cells; *β-TCP* β-tricalcium phosphate; *MBG* mesoporous bioactive glass; *rabMSCs* rabbit mesenchymal stem cells; *MRSA* methicillin-resistant *Staphylococcus aureus*; *PLA* polylactic acid; *PDA* polydopamine; *HMP* heparin methacrylamide microparticles; *BMP-2* bone morphogenetic protein 2; *VEGF* vascular endothelial growth factor; *MVFs* microvascular fragments; *Ref.* references

## Four-dimensional biomaterials

Although the emergence of 3D biomaterials and 3D bioprinting technologies was perceived as dramatic breakthroughs in regenerative medicine, we should not blind ourselves to the limitations of existing biomaterials. Natural tissue regeneration is a highly dynamic and extremely sophisticated process that requires the participation of various biomolecules, cells, and extracellular matrix components in a sequential and precisely controlled manner. Moreover, dynamically reconfigurable microarchitectures with alterable functions are also needed to provide diverse physical and functional support during different phases of tissue conformation. Most traditional biomaterials, however, are designed as inanimate and static substitutes, which fail to cater to the highly dynamic and constantly evolving process of tissue regeneration. To circumvent these limitations, four-dimensional (4D) biomaterials were proposed as the new-generation solution for tissue reconstruction, integrating the conception of time as the fourth dimension [5]. In brief, 4D biomaterials are capable of undergoing self-transformation in the form of shape or functionality upon exposure to predetermined stimuli (e.g., temperature, humidity, osmotic pressure, light, magnetism, electric, and mechanical stimulation) [252]. The dynamic self-remodeling capability and tunable stimuli-responsiveness of 4D biomaterials allow precise real-time control over the hierarchical architecture and functional transformation of complex biomimetic tissue surrogates, exhibiting unprecedented potential within the field of tissue engineering. According to the patterns of stimuli-responsiveness, 4D bioprinted materials could be roughly categorized as materials based on shape-transformation mechanisms and those based on functional transformation mechanisms.

The shape-transformation capability of 4D biomaterials has been extensively investigated, whereas the predetermined stimuli can be further subdivided into physical stimuli (e.g., temperature [253–260], humidity [259, 261], light [262–264], electricity [265], magnetism [266, 267], and acoustic waves [268]), chemical stimuli (e.g., pH value [269, 270] and certain ions [271–274]), and biological stimuli (e.g., cell traction force [275] and enzymes [276]). With regard to bone tissue reconstruction, a variety of thermoresponsive injectable hydrogels, which fit the broad definition of 4D biomaterials, were designed to cater to the need for customized bone defect repair [277–280]. These injectable hydrogels fill in the defects or cracks and undergo sol–gel transition under preset temperatures to achieve dynamic and seamless integration with bone structure, exhibiting distinct superiority in repairing irregularly shaped bone defects. In addition, diverse mineral components (e.g., hydroxyapatite, biphasic calcium phosphate, and mesoporous bioactive glass) were incorporated into thermoresponsive injectable hydrogels to promote the loading-bearing capacity and biological performance of the hybrid hydrogels. For example, hybrid thermoresponsive hydrogels comprising hyaluronic acid-g-chitosan-g-poly(*N*-isopropylacrylamide) and biphasic calcium phosphate exhibited promoted bioactivity and osteogenic capacity, which is corroborated by improved cell propagation, elevated ALP activity, upregulated gene expression of bone formation markers, accelerated calcium deposition rates, and efficient osteoid formation in a subcutaneous implantation model [277]. In addition to hydrogels, biocompatible polymer scaffolds with tunable thermoresponsive shape memory effects were fabricated using polycaprolactone triol and castor oil [281]. The smart polymer scaffolds exhibited satisfactory mechanical integrity along with superior biocompatibility and osteoinductivity. Full shape recovery of the deformed polymer scaffolds could be achieved at the recovery temperature, which may provide us with a less invasive strategy for implantation of biomimetic bone grafts.

**Fig. 11:**
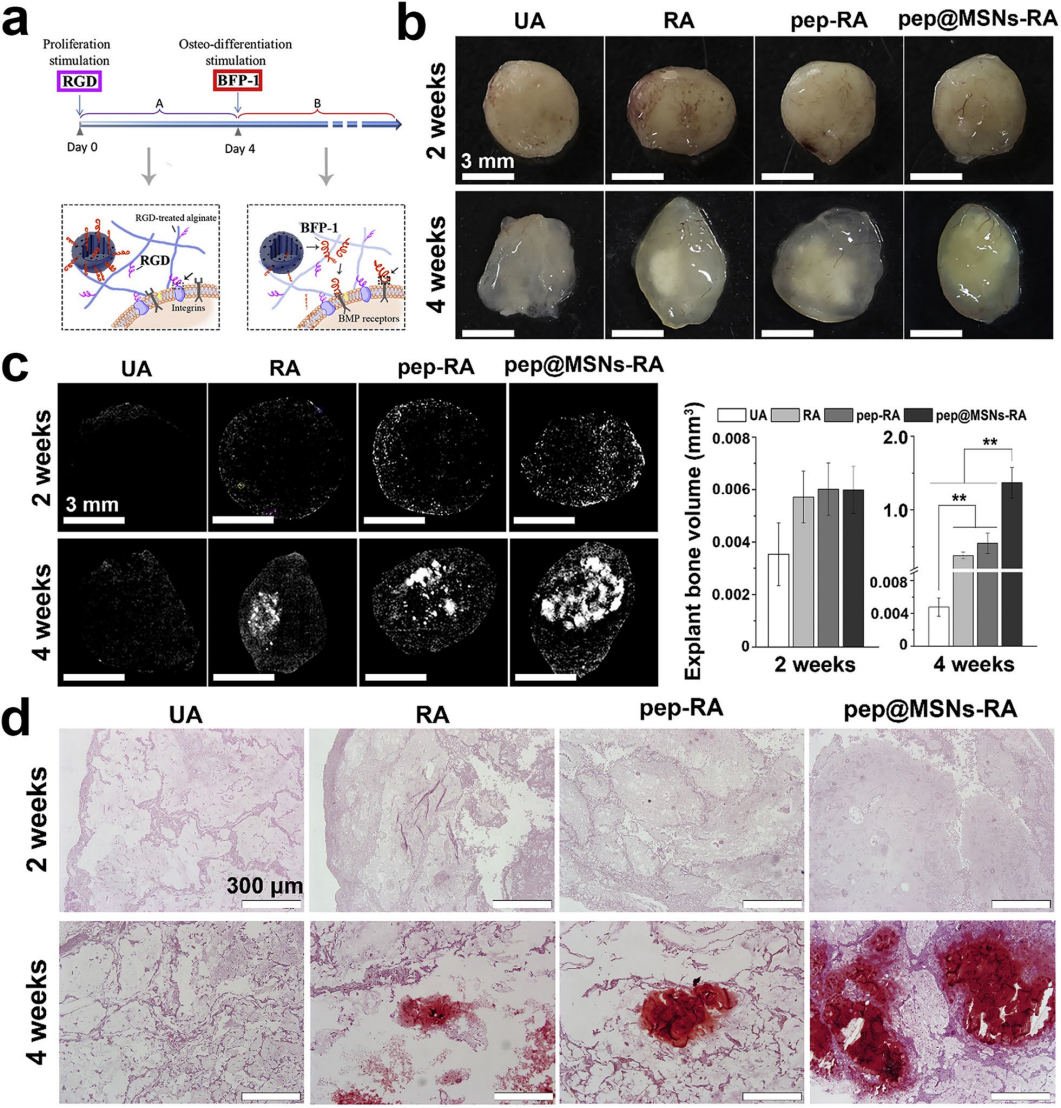
In vivo bone regeneration performance of different hMSCs-loaded hydrogel-based release systems in the mouse subcutaneous pocket model. **a** Schematic illustration of the multifunctional hydrogel-based release system. RGD and BFP-1 incorporated in the hybrid system promote hMSC adhesion and osteogenic differentiation, respectively. **b** Gross view of the implants from different groups after 2 weeks and 4 weeks of implantation. **c** Micro-CT reconstructed images of calcium mineralization within the hydrogels and the corresponding quantitative analysis of bone volume after 2 weeks and 4 weeks of implantation. (Symbols: **, statistical significance with p < 0.01) **d** Alizarin red S staining of the implants from different groups after 2 weeks and 4 weeks of implantation. (*UA* untreated alginate hydrogel; *RA* RGD-treated alginate hydrogel; *pep-RA* BFP-1-incorporated RA; *pep@MSNs-RA* BFP-1-loaded mesoporous silica nanoparticles incorporated into RA) [Panels **a**–**d** are from Luo et al. [282], reprinted with permission. Copyright © 2018 Elsevier]

Regarding functional transformation of 4D biomaterials, the tunable functionality of 4D tissue substitutes enables them to manipulate cell behavior and cell fate in an evolving manner, resembling the functional transition feature of native tissues. One straightforward example to illustrate the concept of functional transformation is a dual-peptide loaded hybrid hydrogel designed by Luo et al. [282]. In this hybrid system, RGD and bone forming peptide-1 (BFP-1)-loaded mesoporous silica nanoparticles were incorporated into the alginate-based hydrogel to form a multifunctional platform for sequential functioning of diverse biomolecules. After MSC adhesion and proliferation were promoted by RGD, BFP-1 loaded on mesoporous silica nanoparticles, which followed a long-term sustained release pattern, started to play a role in guiding MSC differentiation toward the osteogenic lineage (Fig. 11). The favorable osteoinductive effect of the hybrid hydrogel was corroborated in the mouse subcutaneous pocket model. It is also worth mentioning that piezoelectric materials (e.g., barium titanate), which are capable of generating electric charges under mechanical stress, provide a new paradigm for bone tissue reconstruction. In response to the stimulation of applied mechanical stress, piezoelectric biomaterials harness the mechanical force to create an electrical microenvironment, which exerts a favorable influence on cell propagation, osteogenic differentiation, and mineralization of the bone matrix [283]. The exceptional piezoelectric effect of piezoelectric biomaterials presented us with a new strategy for achieving functional transformation of 4D bioprinted bone substitutes during the post-bioprinting stage. Another great example to elaborate the concept of functional transformation of 4D biomaterials is the aforementioned BP-reinforced BG scaffolds (BP-BG) [149]. There is no doubt that BP nanosheets should be categorized as two-dimensional biomaterials according to their spatial size in each dimension. However, the multi-functional characteristics and the spontaneous biodegradation process of BP nanosheets make them conform to the broad definition of 4D biomaterials with functional transition capability. Specifically, the superior photothermal conversion capability of BP nanosheets endows BP-BG with remarkable efficiency in eradicating osteosarcoma under NIR irradiation. Once the photothermal therapy is accomplished and healthy bone tissue starts to regenerate, abundant ${\mathrm{PO}}_{4}^{3-}$ released from the biodegradation process of BP nanosheets could rapidly extract ${\mathrm{Ca}}^{2+}$ ions to form calcium phosphate, which would significantly facilitate the process of biomineralization and bone regeneration. Importantly, while focusing on bone regeneration, we should never neglect the great essence of vascularization and neurotization during the process of bone tissue reconstruction. Several 4D fabrication strategies have been exploited to facilitate vascularization and nerve regeneration in complex tissue engineering, which are particularly inspiring for the reconstruction of large bone defects [284, 285].

Although 4D biomaterials and 4D fabrication technology are still in their infancy, we have already been excited by the unpredictable potential they have revealed. The 4D fabrication technique offers precise spatiotemporal control over the hierarchical microstructure and functionalities of the fabricated tissue substitutes after implantation in coordination with the dynamic process of tissue regeneration. The increased interest in 4D biomaterials opened the path to manufacture biomimetic tissue surrogates with excellent self-remodeling and functional transition capability, which is sure to garner considerable attention in regenerative medicine.

## Conclusions and future perspectives

Based on the size in each dimension, biomaterials utilized within the field of bone regeneration are categorized as zero-dimensional, one-dimensional, two-dimensional, and three-dimensional biomaterials. The distinct dimensional geometry of each category of biomaterials (i.e., high surface-to-volume ratio of zero-dimensional biomaterials, high length-to-diameter ratio of one-dimensional biomaterials, high diameter-to-thickness ratio of two-dimensional biomaterials, hierarchical spatial structure of three-dimensional biomaterials) endows them with unique chemophysical properties and biological performance. Biomaterials from different dimensional categories are usually integrated as hybrid biomaterials, so as to take advantage of each component to provide synergistic effects on supporting cell proliferation and facilitating the process of bone tissue regeneration. Surface modification with chemical groups and functionalization with a variety of biomolecules (e.g., RGD and growth factors) is also an effective method to promote the biocompatibility and bioactivity of biomaterials. Moreover, the rise of 3D bioprinting techniques opened the path for manufacture of cell-laden tissue-like constructs with predesigned structures, whereas the emergence of 4D fabrication technology made it feasible to manipulate the remodeling and functional transformation of bioprinted bone substitutes during the post-bioprinting stage to coordinate with the highly dynamic process of bone reconstruction. Another trend in the design of biomaterials is to endow them with multifunctionality. Biomaterials with superior tumor eradicating capacity and excellent osteoinductivity could serve as stepwise countermeasures for tumor invasion in bone tissues. Infected nonunion, which still represents thorny challenges for orthopedic surgeons, may also be completely conquered by the application of biomaterials with potent antibacterial property and osteoinductive capability.

Despite numerous studies focusing on bone regeneration, autografts still serve as the “gold standard” treatment in cases of bone defects, especially critical-sized defects. The slow clinical translation of the present biomaterials may be ascribed to several factors, such as relatively low bioactivity, relatively uncontrolled degradation rates, deficiency of sophisticated microstructure (e.g., Haversian canals and microvascular circulation), and the potential immunogenicity of cell-laden materials. It is also worth mentioning that neovascularization and neurotization are two essential processes coupled with bone regeneration, which deserve more attention during the design of biomimetic bone grafts. Herein, we anticipate that 3D and 4D bioprinting technology will be more extensively exploited for fabricating custom-tailored living bone substitutes with dynamic shape adaptation and functional transition capability. More strategies will be proposed to induce synchronous or sequential stimulation of vascularization and neurotization during the process of bone regeneration. Due to the extraordinary bioactivity and regenerative potential of native host cells, biomaterials capable of recruiting diverse host cells will also become the focus of regenerative medicine.
